# Toward oral nanomaterial-based drug delivery systems for hepatocellular carcinoma therapy: evidence mapping, route-specific validation, and translational challenges

**DOI:** 10.3389/fphar.2026.1854700

**Published:** 2026-07-01

**Authors:** Yang Fu, Yuanxin Ge, Shixiong Yi, Qifeng Peng, Heng Jiang, Jie Zhou

**Affiliations:** Department of Rehabilitation, Chongqing Traditional Chinese Medicine Hospital, Chongqing, China

**Keywords:** clinical translation, evidence mapping, gut–liver axis, hepatocellular carcinoma, nano-drug delivery systems, oral drug delivery, oral-route validation, pharmacokinetics

## Abstract

Hepatocellular carcinoma (HCC) remains a major cause of cancer-related mortality worldwide, and current systemic therapies are limited by advanced-stage diagnosis, dose-limiting toxicity, drug resistance, and incomplete response rates. Oral nano-drug delivery systems (nano-DDS) are being explored as patient-friendly platforms to improve gastrointestinal protection, intestinal absorption, and hepatic exposure of anticancer agents. However, the evidence base remains uneven: only a minority of HCC nano-DDS studies have been validated through oral administration, whereas many mechanistically important studies rely on intravenous, other parenteral, or *in vitro* models. To avoid overstatement, this review maps the literature according to route of administration, model relevance, comparator choice, pharmacokinetic reporting, and translational readiness. We synthesize design strategies for polymeric, lipid-based, inorganic, biomimetic, stimulus-responsive, ligand-targeted, magnetic, natural product-loaded, and microbiome-modulating systems, while distinguishing direct oral evidence from non-oral mechanistic evidence. We further emphasize practical requirements for clinical translation, including clinically meaningful comparators such as marketed oral formulations, fed/fasted and portal pharmacokinetics, orthotopic and fibrotic/cirrhotic HCC models, long-term hepatotoxicity and immunotoxicity testing, gut microbiome safety assessment, manufacturing reproducibility, and minimum characterization packages under biorelevant gastrointestinal conditions. Rather than presenting oral nano-DDS as a mature therapeutic class, this review frames the field as a promising but incompletely validated area that requires route-specific validation and standardized go/no-go criteria before clinical development for HCC.

## 1 Introduction

Hepatocellular carcinoma (HCC) accounts for approximately 75%–85% of all primary liver cancers, with an estimated 866,000 new cases and 759,000 deaths globally in 2022 according to the latest GLOBOCAN estimates (Sung et al., 2021; Bray et al., 2024), making it the sixth most commonly diagnosed malignancy and the third leading cause of cancer-related mortality worldwide. The alarming incidence–mortality ratio reflects the aggressive biological behavior of HCC and the limitations of current therapeutic interventions. Major etiological risk factors include chronic hepatitis B virus (HBV) and hepatitis C virus (HCV) infection, metabolic dysfunction-associated steatotic liver disease (MASLD), previously termed NAFLD/NASH, aflatoxin exposure, excessive alcohol consumption, and metabolic syndrome (Llovet et al., 2021a; Singal et al., 2023). Notably, MASLD has become the fastest-growing cause of HCC globally, with incidence increasing by approximately 150% from 2010 to 2019, reflecting the global obesity epidemic and necessitating urgent attention to this evolving etiological landscape (Huang et al., 2022). Despite advances in surveillance programs and diagnostic imaging, more than 60% of HCC patients are diagnosed at intermediate or advanced stages, precluding curative surgical options (Villanueva, 2019).

Current therapeutic strategies for HCC encompass surgical resection, liver transplantation, radiofrequency ablation (RFA), transarterial chemoembolization (TACE), and systemic pharmacotherapy (Reig et al., 2022). Sorafenib, the first FDA-approved multi-kinase inhibitor for advanced HCC, extended median overall survival by approximately 2.8 months compared with placebo in the landmark SHARP trial (Llovet et al., 2008). Subsequently, lenvatinib demonstrated non-inferiority to sorafenib as first-line therapy, while atezolizumab combined with bevacizumab established a new standard of care in the IMbrave150 trial (Finn et al., 2020; Cheng et al., 2022). More recently, the HIMALAYA trial demonstrated the superiority of the Single Tremelimumab Regular Interval Durvalumab (STRIDE) regimen over sorafenib, with 4-year overall survival data confirming durable benefit (Abou-Alfa et al., 2022; Sangro et al., 2024). The CheckMate 9DW trial further expanded the immunotherapy landscape, demonstrating significant overall survival improvement with nivolumab plus ipilimumab over sorafenib or lenvatinib monotherapy (Yau et al., 2025). Nevertheless, systemic therapies are associated with substantial adverse effects—including hand–foot skin reactions, diarrhea, hypertension, hepatotoxicity, and immune-related adverse events—which severely compromise patient quality of life and treatment adherence (Cheng et al., 2009). Furthermore, the objective response rates of current first-line regimens remain modest, approximately 27%–30% for the atezolizumab–bevacizumab combination, and reliable predictive biomarkers for immunotherapy response remain elusive (Greten et al., 2023; Llovet et al., 2022), underscoring the urgent need for novel therapeutic strategies with improved efficacy and tolerability profiles.

Oral administration represents the most patient-preferred route of drug delivery owing to its non-invasiveness, ease of self-administration, improved compliance, and reduced healthcare costs (Thanki et al., 2013). However, oral delivery of anticancer agents to hepatic tumor sites presents formidable challenges, including harsh gastrointestinal (GI) conditions, extensive first-pass hepatic metabolism, poor aqueous solubility of most chemotherapeutic agents, P-glycoprotein (P-gp)-mediated efflux, and the biological complexity of the tumor microenvironment (TME). Nano-drug delivery systems (nano-DDS) have emerged as an advanced strategy to overcome these barriers. By encapsulating therapeutic agents within nanoscale carriers, typically 10–200 nm in diameter, oral nano-formulations can protect drugs from GI degradation, enhance intestinal absorption, exploit first-pass metabolism as a targeting advantage for hepatic delivery, achieve controlled drug release, and potentially improve tumor accumulation (Mitchell et al., 2021; Patra et al., 2018). It should be noted that the enhanced permeability and retention (EPR) effect, while extensively demonstrated in preclinical rodent models, has been increasingly questioned in clinical settings. The fundamental delivery bottleneck of cancer nanomedicine has been quantitatively and mechanistically characterised by two landmark studies. First, a multivariate meta-analysis by Wilhelm et al. of 117 nanoparticle tumour-delivery studies published between 2005 and 2015 reported a median delivery efficiency of only 0.7% of the injected dose, with negligible improvement across a decade of formulation engineering (Wilhelm et al., 2016). Importantly, this 0.7% statistic is derived overwhelmingly from intravenously administered, preclinical murine studies—a subsequent reanalysis by Price et al. confirmed that 120 of 136 underlying datasets were xenograft models and every dataset used a single intravenous dose in tumour-bearing mice, and applying classical pharmacokinetic metrics (AUCtumor/AUCblood) to the same studies yields a median relative tumour delivery 113-fold higher than the %ID benchmark (Price et al., 2020). Its applicability to oral nano-DDS scenarios—in which absorption, first-pass hepatic exposure and portal-vein routing differ fundamentally from intravenous bolus pharmacokinetics—therefore remains an open question. Second, Sindhwani et al. provided a mechanistic explanation for the inefficiency captured by Wilhelm’s headline statistic: using four mouse tumour models, three human tumour types, mathematical simulation and two complementary imaging modalities including a “zombie” fixed-vessel control, they demonstrated that up to 97% of nanoparticles enter solid tumours via an active transcellular pathway through endothelial cells, rather than through passive inter-endothelial gaps (Sindhwani et al., 2020). While Sindhwani’s findings derive from rodent and xenograft models and may not extrapolate quantitatively to the fenestrated sinusoidal vasculature of cirrhotic human HCC, together with Wilhelm’s quantification they reframe the rationale for cancer nano-DDS design. Accordingly, the extent to which EPR-mediated passive targeting contributes to oral nano-DDS efficacy in human HCC requires careful re-evaluation. Recent advances in smart nanoparticle design have further expanded the sophistication of these systems, enabling spatiotemporally controlled drug release in response to specific physiological or external stimuli (Sun et al., 2023).

The oral–hepatic axis provides a biologically plausible opportunity for liver-directed delivery because intestinally absorbed drugs and some nanoparticles may enter the portal venous circulation before systemic dilution. Nevertheless, this pathway should be interpreted cautiously. Portal entry does not automatically translate into selective HCC accumulation, because the absorbed fraction may enter lymphatic rather than portal transport, may be rapidly cleared by Kupffer cells, or may be diverted by cirrhosis-associated portal hypertension and portosystemic shunting. Accordingly, the term “oral liver-targeted nano-DDS” is used in this review to describe a design objective rather than a clinically proven targeting outcome unless supported by portal-vein pharmacokinetics, liver-to-plasma exposure ratios, and tumor-to-normal-liver accumulation data.

For this reason, future oral HCC nano-DDS studies should stratify animals and patients by hepatic functional reserve, fibrosis stage, and portal hemodynamic status whenever possible. In experimental studies, portal-vein sampling, liver-to-plasma AUC, tumor-to-normal-liver concentration ratios, and Kupffer-cell uptake measurements should be reported together; liver accumulation alone should not be interpreted as tumor targeting.

Several recent reviews have addressed nanomedicine for liver cancer or oral drug delivery in general, but the present review focuses specifically on the evidence gap between attractive oral-delivery concepts and experimentally validated oral HCC nano-DDS. We therefore organize the literature by route of administration, strength of oral validation, model relevance, comparator choice, and translational readiness. This framing is intended to prevent overinterpretation of non-oral mechanistic studies while still extracting design principles that may inform future oral HCC nano-DDS development.

## 2 Literature search strategy

### 2.1 Search strategy

This structured narrative review was conducted through a literature search of PubMed, Web of Science, Scopus, and Google Scholar. The core search string was: (“oral drug delivery” OR “oral nanoparticle” OR “oral nanomedicine” OR “oral nano-drug delivery system” OR “oral nano-DDS”) AND (“hepatocellular carcinoma” OR “liver cancer” OR “HCC”) AND (“nanoparticle” OR “nano-formulation” OR “nanocarrier” OR “nano-DDS” OR “nanomedicine”). Supplementary searches used the following targeted strings: (“gut–liver axis” OR “gut microbiota”) AND (“HCC” OR “liver cancer”) AND (“nanoparticle” OR “nanomedicine”); (“exosome” OR “extracellular vesicle” OR “biomimetic nanoparticle”) AND (“oral delivery” OR “gastrointestinal delivery”) AND (“liver” OR “HCC”); (“probiotic” OR “engineered bacteria”) AND (“cancer” OR “tumor”) AND (“oral delivery”); and (“sorafenib” OR “lenvatinib” OR “paclitaxel” OR “docetaxel”) AND (“oral nanoformulation” OR “nanocrystal” OR “SNEDDS” OR “solid lipid nanoparticle”). The search was restricted to English-language articles published from January 2008 to March 2026, with emphasis on studies published within the last 5 years. Inclusion criteria were: (i) nanoparticle-based formulations experimentally tested or explicitly proposed for HCC/liver cancer therapy; (ii) studies providing route-of-administration information or mechanistic data relevant to oral delivery; and (iii) peer-reviewed original research, clinical reports, regulatory guidance, or high-quality methodological reviews. Exclusion criteria were: (i) Not relevant to HCC/liver cancer; (ii) Not nanocarrier-based; (iii) λReview/editorial, no primary data; (iv) Insufficient nanocarrier characterization; (v) Network pharmacology or molecular docking only; (vi) Single-concentration *in vitro*, no dose–response.

**FIGURE 1:**
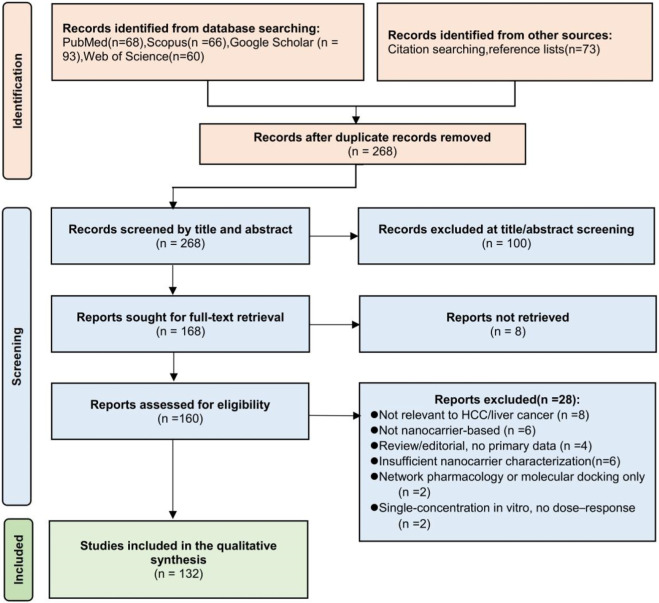
PRISMA-style flow diagram summarizing record identification, duplicate removal, title/abstract screening, eligibility assessment, route-of-administration audit, and final inclusion of oral, parenteral mechanistic, and in vitro-only studies.

A PRISMA-style screening summary was added for transparency (Figure 1).

### 2.2 Scope transparency: oral versus non-oral studies

A transparent evidence hierarchy was applied throughout the review. Because many influential HCC nanomedicine studies were not performed via the oral route, we avoid treating all nano-DDS data as equivalent. Direct oral HCC evidence is discussed as feasibility evidence; oral studies in non-HCC models are treated as absorption or platform evidence; parenteral HCC studies are used only to support mechanistic concepts such as receptor targeting, stimulus-responsive release, or combination rationale; and in vitro-only studies are considered hypothesis-generating. This distinction is especially important for stimulus-responsive systems, ligand-targeted systems, biomimetic platforms, and magnetic theranostic concepts, where oral-route validation remains sparse.

To maintain scientific rigor and transparency, the following conventions are used: (i) oral *in vivo* HCC/liver-tumor studies are described as direct oral evidence; (ii) oral studies in non-HCC disease contexts are described as platform or absorption evidence; (iii) intravenous, intraperitoneal, or other parenteral studies are explicitly annotated as non-oral mechanistic evidence; and (iv) in vitro-only systems are not used to support claims of *in vivo* oral efficacy. This evidence-level framework is formalized in Table 1 and applied throughout the review.

**TABLE 1:** Evidence-level framework used to interpret oral and non-oral nano-DDS studies in this review.

Evidence level	Study type	Interpretation in this review
Level A	Oral *in vivo* HCC or liver-tumor model	Direct evidence for oral HCC nano-DDS feasibility; still requires clinically relevant comparators and orthotopic/fibrotic validation
Level B	Oral *in vivo* non-HCC model	Supports GI stability, absorption, lymphatic/portal transport, or platform feasibility but does not prove HCC efficacy
Level C	Parenteral HCC model	Mechanistic evidence for targeting, TME-responsive release, imaging, or combination therapy; oral translatability remains unproven
Level D	In vitro-only system	Hypothesis-generating evidence for uptake, cytotoxicity, release kinetics, or receptor binding; not sufficient for oral efficacy claims

## 3 Physiological barriers and the oral–hepatic axis

**FIGURE 2:**
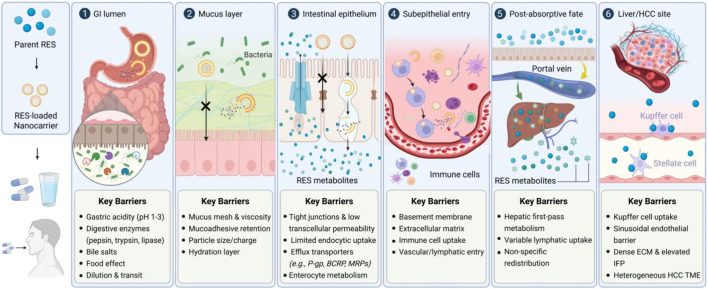
Physiological barriers along the oral–hepatic delivery cascade for HCC-targeted nano-DDS.

The successful delivery of oral nano-formulations to hepatic tumor sites necessitates navigating a complex cascade of physiological barriers, from the oral cavity to the liver parenchyma (Figure 2). Understanding these barriers is prerequisite for the structured design of effective oral nano-DDS for HCC. This section delineates the major physiological impediments and the unique opportunities afforded by the oral–hepatic anatomical connection.

### 3.1 Gastrointestinal pH gradient

The GI tract presents a dramatic pH gradient, ranging from pH 1.0–3.5 in the gastric lumen, pH 6.0–6.5 in the duodenum, pH 7.0–7.4 in the terminal ileum, and pH 5.5–7.0 in the colon (Hua et al., 2015). This highly acidic gastric environment is particularly destructive to protein-based therapeutics, peptide drugs, and certain chemotherapy agents including doxorubicin and sorafenib, which undergo acid-catalyzed degradation (Wang et al., 2020a). The design of pH-responsive nanocarriers that remain intact in acidic gastric conditions (pH < 3.0) but undergo controlled disassembly at intestinal or hepatic pH represents a cornerstone strategy for oral HCC nano-formulations. Enteric coating materials such as Eudragit® L100 with a dissolution threshold of pH 6.0 and S100 with a dissolution threshold of pH 7.0 have been widely employed to protect nano-payloads during gastric transit (Thakra et al., 2013). However, it should be noted that inter-individual and intra-individual variability in gastric pH—particularly in HCC patients with portal hypertensive gastropathy or concurrent proton pump inhibitor use—may significantly affect the performance of pH-dependent release systems, a factor that is frequently overlooked in current preclinical studies.

### 3.2 Intestinal mucus barrier

The intestinal mucus layer, a viscoelastic gel composed primarily of densely glycosylated mucin 2 (MUC2) glycoproteins, serves as the first line of defense against luminal pathogens and xenobiotics (Ba et al., 2018). In the small intestine, the mucus layer is approximately 150–300 μm thick, whereas in the colon it ranges from 110 to 160 μm with a distinct two-layer architecture: an inner firmly adherent layer impermeable to bacteria, and an outer loosely adherent layer colonized by commensal microbiota (Loktionov, 2022). The mesh spacing of the mucus network is approximately 200–500 nm, restricting the diffusion of nanoparticles larger than this threshold (Lai et al., 2009). Conventional cationic nanoparticles, such as unmodified chitosan nanoparticles, exhibit strong mucoadhesive properties that paradoxically trap them in the outer mucus layer, preventing them from reaching the underlying epithelium. PEGylation, i.e., polyethylene glycol coating, has been shown to reduce mucin–nanoparticle interactions, creating “mucus-penetrating particles” (MPPs) with near-unhindered diffusion rates through mucus (Cu and Saltzman, 2009). Nevertheless, it is important to recognize that PEGylation introduces the so-called “PEG dilemma”—while enhancing mucus penetration, PEG surface modification may simultaneously reduce cellular uptake efficiency and has been associated with accelerated blood clearance upon repeated administration due to anti-PEG antibody production (Yang and Lai, 2015). This trade-off necessitates sophisticated surface engineering strategies such as cleavable PEGylation that can temporally decouple mucus penetration from cellular internalization. Recent work on zwitterionic surface coatings and biomimetic virus-inspired surface topologies has offered promising alternatives to PEGylation that may circumvent the anti-PEG immunogenicity problem while maintaining mucus-penetrating capability (Kubiatowicz et al., 2026).

### 3.3 Intestinal epithelial barrier and absorption mechanisms

The intestinal epithelium constitutes a selectively permeable monolayer comprising enterocytes (>80%), goblet cells, Paneth cells, enteroendocrine cells, and M cells overlying Peyer’s patches (Mowat and Agace, 2014). Drug absorption occurs primarily via four pathways: transcellular passive diffusion, paracellular transport through tight junctions, carrier-mediated active transport, and receptor-mediated transcytosis. Nanoparticles are predominantly internalized by enterocytes through clathrin-mediated endocytosis, caveolae-mediated endocytosis, and macropinocytosis, with the specific pathway depending on particle size, surface charge, and functionalization (Rennick et al., 2021). M cells represent a particularly attractive portal for nanoparticle translocation due to their reduced glycocalyx, absence of mucus secretion, and active transcytotic capacity. Nanoparticles of 100–200 nm are preferentially taken up by M cells, providing access to the underlying lymphoid tissue and the mesenteric lymphatic system (Ye et al., 2014). However, M cells constitute only approximately 1% of the intestinal epithelial surface, and their distribution varies considerably between species, necessitating caution when extrapolating M cell-dependent absorption data from rodent models to human applications. Recent studies employing ligand-modified nanoparticles designed to attenuate protein corona adsorption in the gut lumen have demonstrated significantly enhanced gut-to-liver delivery efficiency, highlighting the importance of addressing the *in vivo* corona effect in oral nano-DDS design (Wang J. et al., 2024). Furthermore, the formation of a “bio-corona” on nanoparticle surfaces in the GI lumen—composed of mucins, bile salts, digestive enzymes, dietary proteins, and lipids—is fundamentally distinct from the serum protein corona that forms following intravenous injection, and represents a critically underappreciated determinant of oral nano-DDS performance. The GI bio-corona can alter nanoparticle size, surface charge, targeting ligand accessibility, and cellular uptake efficiency in ways that are difficult to predict from *in vitro* characterization in simplified buffer systems. For instance, adsorption of bile salts and pancreatic lipase onto lipid-based nanocarriers can trigger premature drug release, while mucin adsorption onto PEGylated surfaces may partially negate mucus-penetrating properties. Recent studies have begun to systematically characterize the GI bio-corona using proteomic and lipidomic approaches, but standardized protocols for corona characterization under fed-state and fasted-state GI conditions remain absent, representing a significant methodological gap that limits the predictive value of current preclinical studies for oral nano-DDS.

It is important to note that nanoparticles absorbed across the intestinal epithelium do not uniformly enter the portal venous circulation. Two parallel post-absorptive transport pathways exist: (a) direct entry into the submucosal capillary network draining into the portal vein, which constitutes the predominant route for hydrophilic small-molecule drugs and most polymeric nanoparticles; and (b) uptake into the intestinal lymphatic vessels via M-cell transcytosis or enterocyte-mediated chylomicron association, followed by transport through mesenteric lymph nodes to the thoracic duct and ultimately the systemic venous circulation, thereby bypassing first-pass hepatic metabolism entirely. The lymphatic pathway is particularly relevant for lipid-based nanocarriers, which can associate with endogenous chylomicrons and exploit the intestinal lymphatic transport machinery. The relative contribution of these two pathways to overall nano-DDS absorption is determined by nanoparticle composition, size, lipophilicity, and surface properties, and represents a critical but frequently uncharacterized variable in oral nano-DDS pharmacokinetic studies.

### 3.4 First-pass hepatic metabolism

Following intestinal absorption, drugs that enter the submucosal capillary network are transported through the portal vein, which supplies approximately 75% of hepatic blood flow before systemic distribution (Trefts et al., 2017). For HCC therapy, this first-pass exposure can be leveraged as a potential liver-enrichment mechanism rather than being viewed solely as a bioavailability barrier. However, selective HCC delivery cannot be inferred from portal entry alone. First, a substantial fraction of some lipid-based nanoparticles may enter intestinal lymphatics and bypass the portal vein. Second, portal blood is distributed to normal liver, cirrhotic nodules, Kupffer cells, and tumor tissue rather than exclusively to malignant lesions. Third, many HCC patients have cirrhosis, portal hypertension, and intrahepatic or extrahepatic portosystemic shunts, which can divert portal flow away from hepatic sinusoids and weaken the assumed first-pass advantage. Therefore, future studies should quantify portal-vein exposure, liver-to-plasma AUC, tumor-to-normal-liver AUC, and the effect of fibrotic/cirrhotic hemodynamics before claiming liver or tumor targeting.

### 3.5 Hepatic sinusoidal and tumor microenvironment barriers

Within the liver, nanoparticles encounter the fenestrated sinusoidal endothelium, Kupffer cells, hepatic stellate cells, and the heterogeneous HCC tumor microenvironment (Tsuchida and Friedman, 2017). Although HCC is often described as hypoxic, acidic, ROS-enriched, enzyme-rich, and immunosuppressive (Ringelhan et al., 2018; Yang et al., 2019), these features vary substantially across tumor stage, differentiation grade, etiology, vascularization, fibrosis burden, and prior treatment exposure. As a result, pH-, ROS-, GSH-, enzyme-, or immune-responsive release systems should be interpreted as conditional designs whose performance depends on the patient-specific microenvironment rather than as universally reliable triggers. Orthotopic HCC models on fibrotic or cirrhotic backgrounds are therefore essential for evaluating whether these microenvironmental triggers remain strong enough, spatially accessible enough, and reproducible enough to support selective drug release in clinically relevant disease settings.

### 3.6 P-glycoprotein efflux and multi-drug resistance

P-glycoprotein (P-gp/ABCB1), a transmembrane ATP-dependent efflux pump, is abundantly expressed on both intestinal epithelial cells and HCC cells, actively extruding a broad spectrum of chemotherapeutic agents including doxorubicin, paclitaxel, and sorafenib from the intracellular compartment (Robey et al., 2018). P-gp-mediated efflux reduces both intestinal drug absorption and intracellular drug accumulation in tumor cells, constituting a dual barrier to oral HCC therapy. Nano-encapsulation can circumvent P-gp efflux by mediating endocytotic cellular uptake that bypasses the apical efflux pump, while co-delivery of P-gp inhibitors, such as verapamil, elacridar, or natural compounds like curcumin, within the same nanocarrier represents a synergistic strategy to overcome MDR (Li et al., 2016). However, it should be acknowledged that P-gp is not the sole efflux transporter relevant to oral HCC therapy: breast cancer resistance protein (BCRP/ABCG2) and multidrug resistance-associated proteins (MRPs/ABCCs) also contribute to chemoresistance in HCC and should be considered in comprehensive MDR reversal strategies.

## 4 Design strategies and material selection for oral liver-targeted Nano-DDS

**FIGURE 3:**
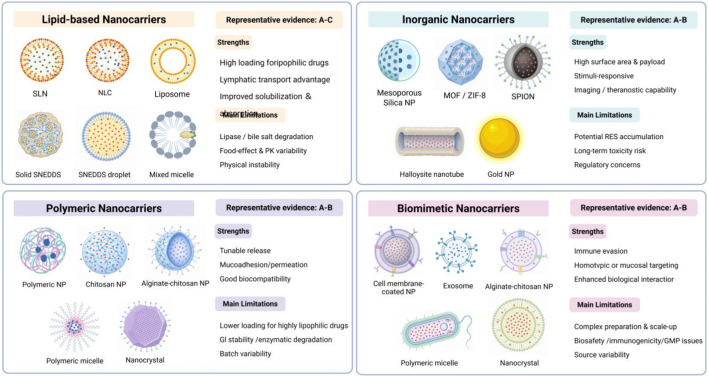
Structural comparison of major oral nano-DDS platforms for hepatocellular carcinoma.

The structured design of oral nano-DDS for HCC therapy requires an integrative approach that simultaneously addresses GI stability, mucosal penetration, intestinal absorption enhancement, hepatic targeting, and tumor-selective drug release. This section outlines the key design principles and commonly employed nanomaterials (Figure 3).

### 4.1 Size, shape, and surface engineering

Particle size is a critical determinant of oral bioavailability, mucus penetration, and hepatic disposition. Nanoparticles in the 50–200 nm range demonstrate optimal intestinal absorption, with particles <100 nm showing the highest transcytosis efficiency across M cells and enterocytes (Banerjee et al., 2016). For hepatic targeting, particles of 100–200 nm can traverse the enlarged sinusoidal fenestrae of HCC vasculature while avoiding rapid renal clearance (<10 nm) and excessive Kupffer cell uptake (>200 nm) (Blanco et al., 2015). Surface charge profoundly influences mucosal interaction: while cationic surfaces enhance mucoadhesion, zwitterionic or neutral PEG-coated surfaces optimize mucus penetration. A charge-switching strategy—negative or neutral surface charge during GI transit transitioning to positive charge in the acidic TME—has emerged as an effective strategy to reconcile these competing requirements (Yuan et al., 2012). Beyond size and charge, nanoparticle shape has recently been recognized as an underappreciated determinant of oral bioavailability: rod-shaped and discoidal nanoparticles demonstrate enhanced intestinal adhesion and margination behavior compared with spherical counterparts, although this parameter remains largely unexplored in oral HCC nano-DDS design and represents a promising avenue for optimization.

### 4.2 Polymeric nanoparticles

Biodegradable polymeric nanoparticles constitute the most extensively investigated platform for oral HCC nano-DDS (Gao et al., 2025). Poly(lactic-co-glycolic acid) (PLGA), approved by the FDA for parenteral use, demonstrates excellent biocompatibility, tunable degradation kinetics, and high drug encapsulation efficiency (Danhier et al., 2012). Chitosan, a cationic polysaccharide derived from chitin deacetylation, enhances intestinal permeability through reversible opening of epithelial tight junctions via interaction with zonula occludens-1 (ZO-1) proteins, and is degraded by colonic microflora-secreted enzymes, providing colon-specific release (Choukaife et al., 2022). Alginate–chitosan polyelectrolyte complex nanoparticles combine the acid-resistance of alginate with the permeation-enhancing properties of chitosan, creating robust oral carriers (Li et al., 2022). Poly(N-isopropylacrylamide) (PNIPAAm) introduces thermoresponsive behavior, enabling temperature-triggered drug release in the hyperthermic HCC microenvironment (Fu et al., 2009). Recent advances in chitosan-coated PLGA nanoparticles have demonstrated enhanced cytotoxicity against human liver cancer cells through improved cellular uptake and sustained intracellular drug release (Alsulays et al., 2024). Despite these advances, the standardized characterization of polymeric nano-DDS stability under physiologically relevant GI conditions, including fed-state bile salt interactions and food matrix effects, remains a critical gap. In a study that directly bridges polymeric nanocarrier design with the P-gp efflux challenge, sorafenib nanocrystals stabilized with Labrasol® (caprylocaproyl polyoxyl-8 glycerides)—a surfactant that simultaneously functions as a P-gp inhibitor—were developed and evaluated via oral administration in rats (Nasr et al., 2025). The Labrasol-stabilized nanocrystals significantly enhanced both Cmax and AUC compared with plain sorafenib, while *in vitro* studies confirmed reversal of multidrug resistance in HepG2/MDR cells through inhibition of P-gp-mediated efflux. This dual-function excipient strategy—combining nanosizing-mediated dissolution enhancement with efflux pump inhibition within a single formulation component—represents a pragmatic and readily scalable design paradigm for oral HCC nano-DDS.

### 4.3 Lipid-based nanocarriers

Lipid-based nano-DDS, including solid lipid nanoparticles (SLNs), nanostructured lipid carriers (NLCs), liposomes, and self-nanoemulsifying drug delivery systems (SNEDDS), offer distinctive advantages for oral HCC therapy due to their biomimetic lipid composition, high drug loading for lipophilic agents, and ability to exploit intestinal lymphatic transport (Trevaskis et al., 2008; Mahmoud et al., 2022). Lymphatic absorption bypasses first-pass hepatic metabolism for drugs that would otherwise be extensively metabolized, while simultaneously enabling mesenteric lymph node-mediated immune modulation—a dual advantage for HCC immunotherapy (Mu and Holm, 2018). Self-assembling bile salt–phospholipid mixed micelles mimic endogenous bile components, facilitating intestinal absorption through bile acid transport pathways (Chen et al., 2009). The SNEDDS platform has been specifically validated for oral sorafenib delivery with quantitative pharmacokinetic evidence. A solid SNEDDS formulation prepared by spray-drying liquid SNEDDS with Aerosil® 200 and PVP K30 markedly reduced sorafenib crystallinity and improved dissolution under simulated gastric and intestinal conditions (Lim et al., 2023). Oral pharmacokinetic evaluation in rats demonstrated a 2.8-fold increase in Cmax (6.1 ± 0.6 μg/mL) and a 4.6-fold increase in AUC (136.1 ± 25.9 h μg/mL) compared with free sorafenib, while the solid form conferred improved storage stability over conventional liquid SNEDDS—addressing a key commercial viability concern. As a complementary non-lipid approach, crystalline solid dispersions (CSDs) of sorafenib using poloxamer 188 as the carrier have demonstrated that steric hindrance imposed by the crystallized polymer network can restrict drug crystal growth, thereby modulating dissolution behavior and improving both oral bioavailability and anti-liver cancer efficacy *in vivo* (Zhang Y. et al., 2025). These studies collectively establish a quantitative oral bioavailability enhancement range of approximately 3–5-fold relative to free drug suspension for sorafenib. Notably, natural lipid nanoparticles derived from plant sources, such as Morus nigra leaves, have demonstrated notable hepatic targeting capability via the oral route, representing a novel biomimetic approach that merits further exploration (Gao et al., 2024). Lipid nanoparticles incorporating ionizable lipids, initially developed for mRNA delivery, are now being repurposed for liver-targeted oral therapeutics, with bile acid-containing formulations showing enhanced hepatic tropism (Chu et al., 2024). A noteworthy limitation of lipid-based nanocarriers is their inherent susceptibility to lipase-mediated degradation in the GI tract and their sensitivity to food effects, which can result in highly variable oral bioavailability—a major concern for chemotherapeutic agents with narrow therapeutic windows. The food effect represents a particularly critical variable: co-administration with a high-fat meal can alter bile salt concentrations, gastric emptying kinetics, intestinal pH, and luminal lipase activity, directly impacting nanocarrier stability and drug release. These factors compound the previously described bio-corona effects, as fed-state GI conditions substantially alter the composition and thickness of the nanoparticle bio-corona. To date, no oral nano-DDS for HCC has been systematically evaluated under both fed-state and fasted-state conditions using biorelevant dissolution media like FaSSIF/FeSSIF. Future preclinical studies should routinely incorporate food effect assessments, as this concern extends beyond lipid-based carriers to all categories of oral nano-DDS.

### 4.4 Inorganic nanoplatforms

Mesoporous silica nanoparticles (MSNs), with their ordered pore structure (2–50 nm), high specific surface area (>1000 m^2^/g), and facile surface functionalization, represent versatile drug reservoirs for oral delivery (Vallet-Regí et al., 2017). The pore architecture enables high drug loading via physical adsorption and provides inherent controlled-release properties through pore-gating mechanisms responsive to pH, enzymes, or redox stimuli (Li et al., 2012). Metal–organic frameworks (MOFs), particularly zeolitic imidazolate framework-8 (ZIF-8), have garnered significant attention as pH-responsive oral carriers due to their stability at neutral and alkaline pH but rapid dissolution in acidic environments (pH < 6.0), enabling gastric protection followed by intestinal drug release (Sun et al., 2012). The versatility of MOF-based smart stimuli-responsive drug delivery systems has been extensively reviewed, with particular emphasis on their programmable degradation kinetics and multi-stimulus responsiveness for cancer therapy (Guo et al., 2025). Superparamagnetic iron oxide nanoparticles (SPIONs) provide magnetic targeting capability combined with MRI contrast enhancement, enabling theranostic applications (Vangijzegem et al., 2019; Li et al., 2026). However, the long-term biosafety of inorganic nanomaterials following chronic oral exposure remains a significant concern: unlike biodegradable polymeric carriers, inorganic nanoparticles may accumulate in hepatic, splenic, and renal tissues over time, with incompletely characterized consequences for organ function and systemic immunity. Rigorous chronic toxicity evaluation, including assessment of hepatic fibrosis induction and gut microbiome perturbation, is imperative before clinical translation of inorganic oral nano-DDS (Docter et al., 2015).

### 4.5 Critical assessment and cross-platform comparison of material strategies

A comparative evaluation of the three principal material classes reveals distinct trade-offs that should guide rational platform selection. Polymeric nanoparticles offer the most mature regulatory pathway and greatest formulation flexibility, with PLGA-based systems benefiting from existing FDA-approved precedents; however, their drug loading capacity for hydrophobic agents is typically limited to 5%–15% w/w. Lipid-based nanocarriers achieve substantially higher loading for lipophilic drugs (up to 30%–50% w/w) and exploit endogenous lymphatic transport, but are inherently susceptible to GI lipase degradation and food effects, introducing pharmacokinetic variability that is particularly problematic for narrow-therapeutic-window chemotherapeutics. Inorganic nanoplatforms provide the most versatile stimulus-responsive architectures and highest surface areas for drug loading, but face the most significant long-term biosafety concerns due to potential tissue accumulation. Critically, no head-to-head comparison of polymeric, lipid-based, and inorganic oral nano-DDS delivering the same therapeutic agent to the same HCC model has been reported, making it impossible to rank-order these platforms by actual oral delivery efficiency. Such comparative studies, employing standardized pharmacokinetic endpoints, should be a priority for the field. Stability testing under biorelevant GI conditions remains critically lacking across all material classes.

A clinically important comparator issue should be emphasized. Many oral nano-DDS studies report large bioavailability gains relative to free drug suspensions, but free suspensions are often poor comparators for clinically used drugs that already employ an optimized marketed oral formulation; reporting fold-improvement only against a crude suspension may therefore overstate the true translational advantage. Future studies should therefore report both formulation-control and clinical-benchmark comparisons.

A further imbalance in the current oral nano-DDS literature is the disproportionate focus on sorafenib as the model drug payload, with lenvatinib—now a co-equal first-line agent for advanced HCC—almost entirely absent from oral nano-formulation studies. As a substrate of both P-gp and BCRP, lenvatinib shares many of the biopharmaceutical challenges that motivate nanocarrier intervention, including food-effect sensitivity and inter-individual pharmacokinetic variability exacerbated by hepatic impairment (Qin et al., 2024). Although numerous nanocarrier platforms have been developed for lenvatinib delivery to HCC, these systems have uniformly employed parenteral administration. The absence of any dedicated oral lenvatinib nano-DDS study is a striking omission, given that lenvatinib is clinically administered as an oral capsule and could benefit substantially from the bioavailability enhancement and first-pass hepatic targeting strategies detailed in this review. We recommend that the field prioritize parallel development of oral nano-formulations for both sorafenib and lenvatinib, enabling cross-drug comparative pharmacokinetic evaluation within the same nanocarrier platform.

## 5 Stimulus-responsive oral nano-formulations for sequential drug release

**FIGURE 4:**
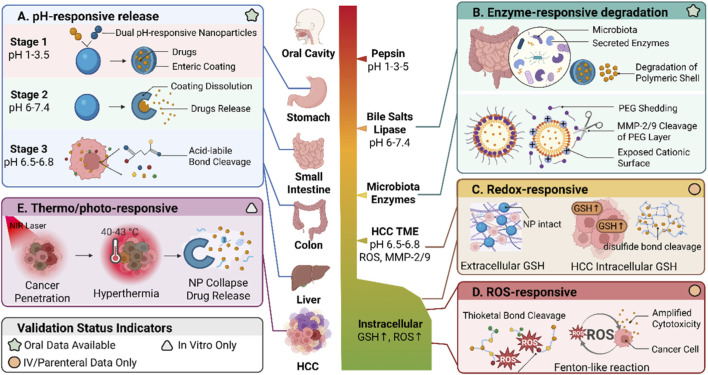
Stimulus-responsive mechanisms exploited by oral nano-formulations for sequential drug release along the oral–hepatic axis.

Stimulus-responsive, often termed “smart,” nano-formulations are designed to exploit endogenous or exogenous triggers for spatiotemporally controlled drug release. In the context of oral HCC therapy, these systems are attractive because they could theoretically remain stable during GI transit and release payloads after reaching hepatic tumor tissue. Nevertheless, trigger responsiveness demonstrated in simplified buffers, cultured cells, or parenteral tumor models should not be assumed to operate with the same magnitude in human HCC after oral administration. The relevant gradients—gastric and intestinal pH, mucus composition, bile salts, redox state, ROS level, enzyme expression, tumor acidity, fibrosis, and immune-cell infiltration—are continuous, dynamic, and patient-dependent rather than fixed binary conditions (Figure 4).

### 5.1 pH-responsive oral nano-formulations

pH-responsive nano-DDS exploit the pH differential between the gastric lumen (pH 1–3), intestinal lumen (pH 6–7.4), and the acidic HCC TME (pH ∼6.5–6.8, as discussed above) to achieve sequential protection, release, and tumor-selective activation (Kanamala et al., 2016). The most common pH-responsive mechanisms include: (i) dissolution of acid-labile enteric coatings, such as Eudragit® and hydroxypropyl methylcellulose phthalate, at intestinal pH; (ii) protonation/deprotonation-induced swelling of polyelectrolyte hydrogels; and (iii) cleavage of acid-labile chemical bonds, including hydrazones, Schiff bases, and acetals, in the acidic TME (Du et al., 2011).

A representative pH-responsive co-delivery system employed calcium carbonate nanoparticles coated with a lipid bilayer for sequential release of Tim-3 siRNA and sorafenib against HCC (Song et al., 2022). The system incorporated sequential pH-responsive behavior: stability under neutral conditions with accelerated disassembly in acidic endosomal compartments. In HCC-bearing mouse models, the co-delivery system significantly enhanced antitumor efficacy compared with single-agent controls, demonstrating the potential of pH-responsive architectures for combination gene–drug therapy in HCC.

In an advance specifically addressing oral delivery, a recent study employed microfluidic technology to encapsulate sorafenib-loaded ZIF-8 nanoparticles within pH-responsive alginate microparticles for oral chemotherapy of HCC (Mirshafiei et al., 2026). The ZIF-8 metal–organic framework core provided pH-triggered drug release in the acidic tumor microenvironment, while the alginate shell conferred gastric protection. This microfluidic approach offered superior batch-to-batch reproducibility compared with conventional preparation methods, addressing a key development concern for oral nano-DDS manufacturing. This ZIF-8@alginate system exemplifies a dual-pH-responsive design paradigm particularly suited for oral HCC delivery: the alginate shell exploits the gastric-to-intestinal pH transition (pH < 3.0 → pH > 6.0) for enteric protection and intestinal release, while the ZIF-8 core exploits the physiological-to-TME pH transition (pH 7.4 → pH < 6.0) for tumor-selective drug liberation, thereby achieving two sequential pH-triggered events within a single nanoplatform. While promising, the reliance on subcutaneous xenograft models for most pH-responsive systems warrants caution. Recent work on pH-responsive polydopamine-paclitaxel-loaded poly(3-hydroxybutyrate-co-3-hydroxyvalerate) nanoparticles has further demonstrated the versatility of pH-triggered release mechanisms for targeted HCC therapy (Wu et al., 2024).

### 5.2 Enzyme-responsive oral nano-formulations

The liver microenvironment and HCC tumors are characterized by the overexpression of specific enzymes, including matrix metalloproteinases (MMP-2, MMP-9), cathepsin B, hyaluronidase, and β-glucuronidase, which can serve as endogenous triggers for site-specific drug release (Kos, 2022). Enzyme-responsive nano-DDS typically incorporate peptide substrates or polysaccharide linkers that are specifically cleaved by target enzymes, destabilizing the nanocarrier structure and liberating the therapeutic payload.

Enzyme-responsive “sheddable stealth” strategies, wherein MMP-cleavable peptide linkers conjugate PEG to drug-loaded nanoparticles, have shown considerable promise for HCC. In these systems, the PEG corona provides mucus-penetrating capability during intestinal transit, while MMP-2 overexpressed in the HCC stroma cleaves the PEG shield upon hepatic accumulation, exposing the underlying cationic surface for enhanced cellular uptake, thereby resolving the aforementioned PEG dilemma through spatiotemporally controlled de-PEGylation. Such sequential responsiveness represents an important design paradigm for oral nano-DDS that must traverse multiple biological barriers.

Polysaccharide-based enzyme-responsive systems exploit colonic microbiota-secreted enzymes for colon-specific release, followed by portal venous absorption and hepatic targeting. For example, chitosan/pectin pH-sensitive coatings have been employed for enzyme-triggered colonic delivery of astragalus polysaccharide nanoparticles, demonstrating that microbiota-secreted enzymes can serve as effective biological triggers for site-specific payload release (Liu et al., 2025). The broader implications of such enzyme-responsive colonic delivery for gut–liver axis modulation and HCC prevention are discussed below. A critical consideration for enzyme-responsive systems is the considerable inter-individual variability in gut microbiome composition and enzymatic activity, which can affect the site and rate of carrier degradation, potentially compromising therapeutic reproducibility.

### 5.3 Redox-responsive oral nano-formulations

The intracellular glutathione (GSH) concentration in HCC cells (2–10 mM) is approximately 100–1000-fold higher than in extracellular fluids (2–20 μM), creating a dramatic redox gradient that can trigger selective intracellular drug release (Saito et al., 2003). Disulfide bonds (–S–S–), diselenide bonds (–Se–Se–), and thioether linkages are commonly incorporated into nanocarrier architectures as GSH-cleavable triggers (Huo et al., 2014). Recent systematic reviews have comprehensively catalogued the diversity of redox-manipulating nanocarriers for anticancer drug delivery, revealing that disulfide-crosslinked systems remain the most extensively validated in preclinical HCC models (Meng et al., 2024).

Galactosylated core–shell nanoparticles with dual pH/GSH sensitivity have been specifically designed for HCC targeting (Qu et al., 2023). Notably, this system has been evaluated *in vitro* only, and *in vivo* oral validation remains pending. These nanoparticles featured an ASGPR-targeting galactose surface ligand coupled with intracellular GSH-triggered disulfide bond cleavage, achieving synergistic selectivity through simultaneous exploitation of receptor-mediated uptake and redox-responsive intracellular release. The dual-responsive system demonstrated significantly enhanced cytotoxicity against HCC cells compared with single-stimulus controls, while exhibiting minimal toxicity toward normal hepatocytes. This work exemplifies the emerging trend toward multi-stimulus-responsive nanocarriers that sequentially exploit the distinct microenvironmental conditions encountered during oral-to-tumor delivery.

### 5.4 ROS-responsive oral nano-formulations

Elevated ROS levels—including H_2_O_2_, superoxide, and hydroxyl radicals—are a hallmark of the HCC TME, driven by mitochondrial dysfunction, hypoxia-reoxygenation, and inflammatory cell infiltration (Lin et al., 2018; Takaki and Yamamoto, 2015). Although the ROS mechanisms characterized in Ref (Lin et al., 2018) were primarily studied in colorectal cancer, the underlying pathways—including mitochondrial dysfunction and inflammatory infiltration—are well conserved across solid tumors. HCC-specific ROS elevation has been independently documented in the context of chronic hepatitis, hepatic iron overload, and mitochondrial dysfunction in cirrhotic livers (Takaki and Yamamoto, 2015). ROS-responsive nanomaterials incorporating thioketal bonds, phenylboronic acid esters, or poly(propylene sulfide) undergo oxidation-triggered degradation in high-ROS environments, enabling tumor-selective drug release (Xu et al., 2016; Hu et al., 2024).

A tumor microenvironment ROS/pH cascade-responsive supramolecular nanoplatform was developed for enhanced HCC therapy (Shi et al., 2024). This system incorporated glycyrrhetinic acid as a liver-targeting ligand and thioketal-based ROS-responsive linkers, achieving cascade-responsive drug release triggered sequentially by the acidic pH and elevated ROS of the HCC microenvironment. Notably, the nanoplatform also exhibited ROS regeneration capability, amplifying the intracellular oxidative stress to potentiate tumor cell killing. In HCC-bearing mouse models, the cascade-responsive system demonstrated substantial tumor growth inhibition with minimal systemic toxicity. The combination of ROS-responsive release with ROS amplification represents a notable strategy, as it addresses the immunosuppressive TME of HCC while simultaneously delivering cytotoxic therapy; however, the potential for excessive ROS scavenging by the responsive moiety to paradoxically attenuate oxidative stress-mediated tumor cell apoptosis deserves further mechanistic investigation.

### 5.5 Thermo-responsive and photo-responsive oral nano-formulations

Thermo-responsive nanocarriers based on polymers exhibiting lower critical solution temperature (LCST) behavior, such as PNIPAAm (LCST ∼32 °C), undergo a hydrophilic-to-hydrophobic phase transition upon mild hyperthermia (40 °C–43 °C), triggering drug release (Schmaljohann, 2006). When combined with photothermal transduction agents such as indocyanine green (ICG), gold nanorods, or polydopamine, externally applied near-infrared (NIR) laser irradiation can induce localized hyperthermia at the hepatic tumor site, simultaneously activating drug release and photothermal ablation—a chemo-photothermal synergistic strategy (Li et al., 2020; Fatima et al., 2024).

The stimulus-responsive mechanism of thermo-responsive nanocarriers has been validated in multiple HCC-relevant contexts. A recent meta-analysis and systematic review of nanoparticle-based photothermal effects in HCC treatment has confirmed the substantial preclinical efficacy of NIR-triggered drug release across multiple studies (Zhang et al., 2026). Importantly, the combination of LCST-mediated drug release with photothermal transduction enables a dual-action mechanism: the same NIR stimulus simultaneously triggers payload liberation and induces localized hyperthermia, creating a temporally synchronized therapeutic response. It should be noted, however, that the clinical feasibility of percutaneous NIR irradiation of hepatic tumors is limited by tissue penetration depth (∼1–2 cm for 808 nm light) and the requirement for precise image-guided tumor localization, which may restrict this approach to superficially located or laparoscopically accessible hepatic tumors. The synergistic therapeutic potential of combining photothermal stimulation with chemotherapy or immunotherapy is further discussed in Section 8.3.

### 5.6 Critical assessment of stimulus-responsive oral nano-formulations

Despite the sophisticated design principles underlying stimulus-responsive oral nano-DDS, the current evidence base remains insufficient for strong clinical claims. Most studies compare responsive systems with free drug rather than with matched non-responsive nanocarriers, making it difficult to determine whether the benefit arises from the stimulus-responsive mechanism or from nano-encapsulation itself. *In vitro* release tests often use idealized pH, GSH, ROS, or enzyme concentrations that do not reproduce the continuous gradients and spatial heterogeneity of human HCC. Moreover, many responsive systems in this section were evaluated by intravenous administration, so their stability during gastric transit, mucus penetration, intestinal absorption, bio-corona formation, and fed/fasted pharmacokinetics remain untested. Future studies should include non-responsive nanocarrier controls, biorelevant GI media, orthotopic/fibrotic HCC models, and oral-versus-parenteral comparisons before claiming clinically meaningful stimulus-responsive oral delivery.

Furthermore, the assumed orthogonality of the five stimulus-responsive mechanisms discussed in Sections 5.1–5.5 may not hold under *in vivo* conditions: for example, the acidic TME pH can accelerate thioketal bond hydrolysis independently of ROS levels, while GSH consumption by redox-responsive moieties may alter intracellular ROS homeostasis, potentially creating unanticipated crosstalk between supposedly independent responsive elements. The extent to which such inter-stimulus crosstalk affects the performance of multi-responsive nanoplatforms remains largely unexplored.

## 6 Ligand-mediated targeted oral nano-formulations for HCC

While the stimulus-responsive strategies exploit endogenous physicochemical gradients for spatiotemporally controlled drug release, active targeting through receptor-ligand interactions provides an additional layer of selectivity. Active targeting through surface conjugation of ligands that selectively bind to overexpressed receptors on HCC cells represents a critical strategy for enhancing tumor-specific drug accumulation and reducing off-target toxicity. The unique receptor expression profile of hepatocytes and HCC cells provides multiple targetable moieties.

### 6.1 Asialoglycoprotein receptor (ASGPR)-Targeted systems

The ASGPR is a C-type lectin receptor exclusively and abundantly expressed on the sinusoidal surface of hepatocytes, approximately 500,000 copies per cell, and is highly expressed in well-differentiated HCC (D'Souza and Devarajan, 2015). It selectively binds terminal galactose and N-acetylgalactosamine residues, mediating rapid receptor-mediated endocytosis with a recycling half-time of ∼15 min. ASGPR-targeted oral nano-DDS have been constructed using galactose, lactose, galactosamine, and asialofetuin as targeting ligands (Huang et al., 2017).

The ASGPR-targeting strategy has been validated across multiple nanocarrier platforms. Galactosylated chitosan-coated nanoparticles showed significantly enhanced hepatoma cell uptake compared with non-galactosylated controls, validating the galactose-mediated endocytosis mechanism (Huang et al., 2023). Co-delivery of ursolic acid and sorafenib using lactobionic acid-modified mesoporous silica nanocomplexes achieved simultaneous inhibition of HCC growth and metastasis through combined ASGPR targeting and dual-drug synergy (Zhao et al., 2017), and the galactosylation strategy has been further confirmed to be broadly applicable across diverse therapeutic payloads including apigenin-loaded PLGA nanoparticles (Ganguly et al., 2021). Despite these encouraging results, the clinical utility of ASGPR-targeting is tempered by a significant biological limitation: ASGPR expression is progressively downregulated in poorly differentiated and advanced-stage HCC, potentially limiting the utility of ASGPR-targeting strategies in the patient populations most in need of effective therapy (D'Souza and Devarajan, 2015). This receptor heterogeneity necessitates companion diagnostic assessment of ASGPR expression status to identify patients most likely to benefit from galactose-targeted nano-DDS.

### 6.2 Transferrin receptor (TfR)-Targeted systems

The transferrin receptor (TfR/CD71) is overexpressed 5–10-fold on HCC cells compared with normal hepatocytes, reflecting the elevated iron demand of rapidly proliferating tumor cells (Tortorella and Karagiannis, 2014). Transferrin (Tf) and anti-TfR antibodies have been employed as targeting ligands for HCC-directed nanoparticle delivery. Tf-conjugated nanoparticles exploit TfR-mediated endocytosis to achieve preferential accumulation in TfR-overexpressing HCC cells, with studies demonstrating significantly enhanced cellular uptake compared with non-targeted controls. The TfR pathway is particularly advantageous for oral nano-DDS because TfR is expressed on both intestinal epithelial cells—facilitating transcytotic absorption—and HCC cells, enabling a dual-barrier targeting strategy that enhances both intestinal permeation and tumor-selective delivery. Recent reviews have highlighted the broader therapeutic potential of transferrin as a nano-based drug delivery signaling molecule across various disease indications, underscoring its versatility as a targeting moiety (Li C. et al., 2024). However, despite the theoretical attractiveness of dual-barrier TfR targeting, quantitative evidence demonstrating enhanced oral-to-tumor delivery efficiency via the TfR pathway in HCC models remains conspicuously absent. The few available studies have demonstrated enhanced cellular uptake *in vitro*, but have not reported oral bioavailability, hepatic accumulation ratios, or tumor growth inhibition data from oral administration experiments. Furthermore, the competitive binding of endogenous transferrin (present at ∼2.5 mg/mL in serum) to TfR may substantially reduce the targeting efficiency of Tf-conjugated nanoparticles following systemic absorption, a factor that has not been adequately addressed in the context of oral nano-DDS.

### 6.3 Folate receptor (FR)-Targeted systems

Folate receptor-α (FR-α) is overexpressed in approximately 40%–60% of HCC specimens, with minimal expression on normal hepatocytes, making it an attractive target for selective drug delivery (Sega and Low, 2008). Folic acid (FA) binds FR-α with nanomolar affinity (Kd ∼0.1 nM) and triggers receptor-mediated endocytosis via caveolae (Zhao et al., 2011). Kabil et al. (2024) developed folic/lactobionic acid dual-targeted polymeric nanocapsules, demonstrating that simultaneous engagement of FR-α and ASGPR substantially enhances selectivity for hepatocellular carcinoma cells over normal hepatocytes. However, it is important to note that folate receptor β (FR-β) expression on tumor-associated myeloid cells in the reticuloendothelial system can influence the biodistribution of folate-functionalized nanoparticles, potentially diverting a fraction of targeted nanocarriers away from tumor cells toward hepatic macrophages.

### 6.4 GPC3-targeted and aptamer-based systems

Glypican-3 (GPC3), a heparan sulfate proteoglycan anchored to the cell membrane via glycosylphosphatidylinositol, is overexpressed in >70% of HCC tumors while virtually absent in normal adult liver tissue, making it a highly specific HCC biomarker (Filmus and Capurro, 2013; Zhang J. et al., 2025). Anti-GPC3 antibodies, nanobodies, and DNA/RNA aptamers have been developed as targeting moieties for HCC-directed nano-DDS. GPC3-peptide-decorated Fe_3_O_4_-GOD nanoparticles have been developed for targeted detection and sequential treatment of small HCC in the complex liver environment (Deng et al., 2022). The GPC3-targeting strategy enabled dual ultrasound/photoacoustic imaging combined with catalytic therapy, demonstrating exceptional selectivity for GPC3-positive tumors. Recent work on an ultrasound-responsive nanodelivery system incorporating GPC3-targeting and sonosensitizer further validated the clinical potential of GPC3-based HCC targeting (Zhang et al., 2024a). The specificity of GPC3 as an HCC target has been further corroborated by parenteral mRNA nanovaccine studies achieving >98% tumor inhibition (Zeng et al., 2026), reinforcing the rationale for incorporating GPC3-targeting ligands into oral nano-DDS platforms. Comprehensive reviews of aptamer-functionalized nanomaterials for HCC have identified over 20 distinct aptamer sequences with validated binding to HCC-associated surface markers, significantly expanding the targeting ligand repertoire beyond traditional antibody-based approaches (Yin et al., 2024). Given the high specificity of GPC3 for HCC over normal hepatocytes, GPC3-targeting approaches may offer a more tumor-selective alternative to ASGPR-targeting for advanced HCC, though the limited tissue penetration of aptamer-functionalized nanoparticles in desmoplastic tumors with dense fibrotic stroma requires further evaluation.

### 6.5 Critical assessment and comparative analysis of targeting strategies

A cross-comparison of the four targeting ligand systems discussed above reveals a fundamental hierarchy of clinical applicability contingent upon HCC differentiation status and disease stage. ASGPR-targeting offers well-validated receptor biology and commercially available ligands (galactose, lactobionic acid) but suffers from progressive receptor downregulation in advanced HCC—precisely the patient population with the greatest unmet therapeutic need. TfR-targeting provides the unique advantage of dual-barrier targeting (intestinal epithelium and tumor cells) but lacks quantitative data on oral-to-tumor delivery efficiency. FR-targeting benefits from nanomolar-affinity ligands but is confounded by FR-β expression on hepatic macrophages, which may divert a substantial fraction of targeted nanocarriers. GPC3-targeting offers the highest HCC specificity but has been evaluated exclusively via parenteral routes; the stability of GPC3-targeting peptides and aptamers under GI conditions remains entirely uncharacterized. Notably, none of the four targeting strategies has been subjected to a head-to-head comparison within the same nanocarrier platform and HCC model, precluding an evidence-based ranking of targeting efficiency. Furthermore, all studies discussed above employed either *in vitro* or subcutaneous xenograft models; the relevance of receptor expression levels and targeting efficiency in orthotopic HCC models on cirrhotic backgrounds remains unknown. Future studies should prioritize: (i) comparative evaluation of multiple targeting ligands within the same delivery platform; (ii) assessment of targeting ligand stability after GI transit; and (iii) validation in orthotopic models that recapitulate the cirrhotic microenvironment characteristic of human HCC (Table 2).

**TABLE 2:** Comparative summary of ligand-targeting strategies for oral HCC nano-DDS.

Targeting strategy	Principal rationale/advantages	Main limitations	Clinical translation implication for oral HCC nano-DDS
ASGPR-targeted systems (galactose, lactose, lactobionic acid)	Well-established hepatocyte/HCC-associated receptor biology; commercially accessible ligands; supports receptor-mediated uptake in well-differentiated HCC.	ASGPR expression may decrease in poorly differentiated or advanced HCC; normal hepatocyte uptake may reduce tumor selectivity; ligand masking after GI transit remains possible	Best suited for ASGPR-positive, well-differentiated tumors; requires receptor-status assessment, oral pharmacokinetics, and tumor-to-normal-liver distribution data
TfR-targeted systems (transferrin or anti-TfR ligands)	Conceptually attractive dual-barrier strategy because TfR may support both intestinal transcytosis and uptake by iron-demanding HCC cells	Endogenous transferrin can compete for receptor binding; quantitative oral-to-tumor delivery data are limited; off-target uptake in proliferating normal tissues remains a concern	Promising but still unproven for oral HCC targeting; should be tested with portal/lymphatic transport studies and matched non-targeted nanocarrier controls
FR-targeted systems (folic acid/folate ligands)	Small, stable, low-cost ligand with high-affinity receptor binding; useful for FR-positive HCC and dual-targeted designs with ASGPR.	FR expression is heterogeneous; FR-β expression on tumor-associated macrophages may divert nanoparticles away from tumor cells; not specific to HCC.	Most appropriate as part of a dual-targeting strategy; requires macrophage-uptake assays, receptor stratification, and oral-route validation
GPC3-targeted systems (antibodies, peptides, aptamers)	Highest tumor-selective rationale among the four targets because GPC3 is strongly associated with HCC and minimally expressed in normal adult liver	Current validation is largely parenteral; GI stability of peptides/aptamers, mucus penetration, and bio-corona effects remain poorly characterized; dense fibrotic stroma may limit penetration	Strongest candidate for tumor-selective HCC targeting, but oral applicability remains early; needs GI-stability testing, orthotopic/cirrhotic models, and GPC3-positive patient selection

## 7 Biomimetic and advanced oral nano-formulations

The ligand-targeting strategies discussed rely on synthetic ligand–receptor interactions that, while effective, face several practical limitations: protein corona masking under GI conditions, immune recognition, and the challenge of maintaining ligand functionality after GI transit. Biomimetic approaches offer a fundamentally different paradigm by leveraging natural biological structures to address these limitations. Biomimetic nano-formulations draw inspiration from natural biological structures and processes to overcome the inherent limitations of synthetic nanocarriers, including rapid immune clearance, limited tumor specificity, and poor GI stability. By harnessing cell membranes, exosomes, and engineered probiotics as delivery platforms or surface coatings, these systems inherit the intrinsic biological functionalities of their source materials—such as immune evasion, homotypic targeting, and mucosal adhesion—while retaining the drug loading and controlled release capabilities of conventional nanocarriers. This section examines three principal biomimetic strategies for oral HCC nano-DDS.

### 7.1 Cell membrane-coated nanoparticles

Biomimetic nanoparticles cloaked with natural cell membranes have emerged as a promising strategy for oral drug delivery, leveraging the inherent biological functions of source cells including immune evasion, prolonged circulation, and homotypic targeting (Fang et al., 2018; He et al., 2024). Cancer cell membrane-coated nanoparticles (CCNPs) inherit the surface antigen repertoire of the source tumor cells, enabling homotypic binding to HCC cells through membrane protein interactions (Guo et al., 2024). Homologous cancer cell membrane-camouflaged nanoparticles have demonstrated targeted drug delivery and enhanced chemotherapy efficacy in HCC (Wu et al., 2023). The cancer cell membrane coating conferred dual advantages: immune evasion from Kupffer cell phagocytosis and homotypic HCC targeting through preserved membrane protein interactions, although the *in vivo* studies were conducted in small cohorts (n = 5–6 per group) typical of early-stage nano-formulation research. The current landscape and future potential of this biomimetic platform have been extensively surveyed in recent reviews (Zhang et al., 2024b). Despite these compelling results, cancer cell membrane-coated nanoparticles face significant translational hurdles: the use of tumor cell-derived membranes raises biosafety concerns regarding residual oncogenic material, batch-to-batch membrane protein variability, and the regulatory complexity of characterizing biologically derived coating materials (Graván et al., 2025). Furthermore, the stability of membrane coatings under the harsh GI environment has not been systematically evaluated.

### 7.2 Exosome-based oral delivery

Exosomes, naturally secreted extracellular vesicles (30–150 nm), possess intrinsic biocompatibility, low immunogenicity, and the ability to traverse biological barriers including the intestinal mucosa and blood–brain barrier (Kalluri and LeBleu, 2020; Li et al., 2025). Milk-derived exosomes have attracted particular attention for oral drug delivery due to their abundant availability, food-grade safety, and demonstrated resistance to gastric and intestinal degradation. These vesicles have shown efficient intestinal absorption and good stability across the GI pH range (Li et al., 2023), and their broad application potential as oral drug delivery systems—including delivery of anticancer drugs and therapeutic nucleic acids—has been independently established (Tian et al., 2023; Timofeeva et al., 2023). Engineered exosomes from diverse cellular sources have been the subject of a recent systematic overview for cancer-targeted therapy, with recent advances in surface functionalization and cargo loading significantly expanding their therapeutic versatility (Zhang et al., 2023). The convergence of exosome biology with HCC diagnostics and targeted drug delivery has been highlighted as a particularly promising area, with exosomes serving dual roles as both therapeutic vehicles and diagnostic biomarkers (Gupta et al., 2024).

### 7.3 Probiotic-based oral delivery systems

Engineered probiotics represent an innovative biological platform for oral drug delivery, combining the natural GI colonization capacity of commensal bacteria with genetically encoded therapeutic functions (Cao et al., 2019). Probiotics can survive gastric acidity, adhere to intestinal epithelium, and modulate the gut microbiome—properties that naturally address several oral delivery barriers. In a representative study, *Escherichia coli* Nissle 1917 was engineered as a probiotic neoantigen delivery vector for precision cancer immunotherapy (Redenti et al., 2024). The engineered probiotics colonized tumors and produced neoantigens that activated tumor-specific T cell responses, achieving significant tumor regression in multiple cancer models. The concept of orally administered engineered bacteria serving as *in situ* therapeutic factories has been further validated by probiotic-guided CAR-T cell targeting strategies (Vincent et al., 2023), though such cell therapy approaches operate through fundamentally different mechanisms than nano-DDS. The broader potential of bioengineered bacteria for cancer immunotherapy has been comprehensively reviewed (Nguyen et al., 2023), while recent work on manganese-engineered *Lactobacillus reuteri* demonstrated enhanced antitumor and immunomodulatory activities for cancer prevention and treatment through oral administration (Cao et al., 2025). While probiotic-based delivery represents a notable convergence of synthetic biology and nanomedicine, significant biosafety and regulatory challenges remain, including horizontal gene transfer risk, uncontrolled bacterial proliferation, and immune-mediated adverse reactions in immunocompromised HCC patients with cirrhosis-associated immune dysfunction (Muhammad et al., 2026).

### 7.4 Critical assessment of biomimetic platforms for oral HCC delivery

While biomimetic nano-formulations represent conceptually appealing approaches, their oral application faces platform-specific challenges warranting dedicated investigation. For cell membrane-coated nanoparticles, the integrity of the membrane coating under the harsh GI conditions detailed in Sections 3.1–3.3 has not been systematically evaluated; it is plausible that membrane proteins essential for homotypic targeting and immune evasion are denatured or stripped during GI transit, potentially eliminating the biomimetic advantage. For exosome-based systems, while milk-derived exosomes demonstrate promising GI stability, the scalable purification of exosomes with consistent size, protein composition, and cargo loading remains a significant manufacturing challenge. For probiotic-based systems, beyond the biosafety and regulatory concerns discussed in Section 7.3, the temporal disconnect between bacterial colonization kinetics (hours to days) and pharmacokinetic requirements of anticancer drug delivery represents an additional fundamental design challenge. These platforms remain at early stages of oral translation, and dedicated oral pharmacokinetic evaluations in clinically relevant HCC models are needed.

## 8 Oral nano-formulations for combination therapy in HCC

The preceding sections have discussed individual design strategies for oral HCC nano-DDS, including stimulus-responsive release, ligand-mediated targeting, and biomimetic platforms. In practice, the heterogeneous nature of HCC necessitates multi-modal therapeutic approaches that integrate multiple strategies within a single nanoplatform. Given the phenotypic and genetic heterogeneity of HCC, monotherapy approaches frequently encounter therapeutic resistance. Combination therapy within oral nano-DDS platforms enables spatiotemporally synchronized multi-modal action, synergistic efficacy, and reduced individual drug doses with consequent toxicity mitigation. As noted in Section 2.2, the majority of combination nano-DDS platforms described here were evaluated via intravenous administration. The translation to oral delivery introduces additional complexity: the differential stability of co-encapsulated agents (e.g., hydrophilic siRNA versus hydrophobic sorafenib) under GI conditions—compounding the previously discussed GI stability challenges—may result in asynchronous release and loss of the intended synergistic ratio at the tumor site. Future oral combination nano-DDS designs should incorporate stability testing under biorelevant GI conditions and validate the maintenance of synergistic drug ratios throughout the oral-to-tumor delivery cascade.

### 8.1 Chemo–immunotherapy co-delivery

The immunosuppressive microenvironment of HCC, characterized by exhausted CD8^+^ T cells, abundant regulatory T cells, and tolerogenic dendritic cells, represents a major obstacle to effective immunotherapy (Pinato et al., 2020; Llovet et al., 2024). Oral nano-DDS co-delivering chemotherapeutic agents with immunomodulators can achieve immunogenic cell death (ICD)-mediated immune activation concurrent with checkpoint blockade, converting immunologically “cold” tumors to “hot” phenotypes (Kroemer et al., 2013). The application of nanoparticles in immunotherapy for HCC has been highlighted in recent literature, with co-delivery of chemotherapeutic agents and immune checkpoint inhibitors or STING agonists identified as a particularly promising strategy (Hu et al., 2023). In a recent preclinical study employing intravenous rather than oral administration, napabucasin was shown to deactivate STAT3 and promote mitoxantrone-mediated cGAS-STING activation for HCC chemo-immunotherapy using a nanoparticle co-delivery platform (Wang L. et al., 2025). While this system was not designed for oral delivery, the mechanistic insights regarding STAT3 inhibition combined with cGAS-STING pathway activation provide a mechanistic framework for designing oral nano-DDS with dual chemo-immunotherapeutic capability. Smart responsive Fe/Mn nanovaccines triggering liver cancer immunotherapy via pyroptosis and cGAS-STING activation have further demonstrated the synergistic potential of metal ion-based immunostimulation combined with nanocarrier delivery (Du et al., 2024).

### 8.2 Gene–drug co-delivery

RNA interference (RNAi) technologies, including small interfering RNA (siRNA) and microRNA (miRNA), have shown considerable potential for silencing oncogenic drivers in HCC (Yuan et al., 2025; Weng et al., 2019). Co-delivery of siRNA with chemotherapeutic agents within oral nanocarriers addresses MDR by simultaneously suppressing resistance-associated genes while delivering cytotoxic payloads. Nanoplatform-based *in vivo* gene delivery systems for cancer therapy have made significant advances, with mesoporous silica-based ternary complexes combining drug payloads with gene silencing agents and hepatocyte-targeting ligands representing a particularly sophisticated approach (Luo et al., 2024). The CRISPR/Cas9 gene editing platform has emerged as a next-generation tool for HCC therapy, with recent work demonstrating successful somatostatin receptor-targeted polymeric nanoplatform delivery of CRISPR/Cas9 components for synergistic hepatocellular carcinoma treatment. Notably, this system was administered intravenously, and oral adaptation remains a significant challenge (Zhang S. et al., 2025). The adaptation of such gene-editing nano-DDS for oral administration remains a significant challenge, primarily due to the susceptibility of CRISPR components to GI degradation. A persistent challenge for oral gene–drug co-delivery is maintaining siRNA stability and bioactivity throughout GI transit, as nucleases in the intestinal lumen can rapidly degrade unprotected RNA cargo; the development of chemically modified siRNA, such as 2′-O-methylation and phosphorothioate backbone modifications, compatible with nanocarrier encapsulation represents an important area for optimization.

Encouragingly, oral polymeric nanoparticle-mediated nucleic acid delivery has been experimentally demonstrated in a cancer therapy context. Multifunctional trimethyl chitosan nanoparticles co-loaded with survivin-targeting shRNA and paclitaxel were administered by oral gavage in a murine colon cancer model, achieving functional gene silencing in tumor tissue and synergistic antitumor efficacy (Han et al., 2014). While this study was conducted in a colorectal rather than HCC model, it provides critical proof-of-concept that orally delivered polymeric nanoparticles can protect nucleic acid cargo through GI transit and achieve functional gene knockdown at distal tumor sites—a milestone for the feasibility of oral gene–drug co-delivery strategies applicable to HCC.

### 8.3 Chemo-photothermal synergy

Photothermal therapy (PTT) induces localized hyperthermia (>42 °C) that directly kills tumor cells through protein denaturation and membrane disruption, while simultaneously enhancing tumor vascular permeability, drug diffusion, and cellular uptake of co-delivered chemotherapeutics (Liu et al., 2019). The chemo-photothermal synergy is particularly promising for HCC, as the liver is accessible to percutaneous NIR irradiation under ultrasound guidance (Da et al., 2021). Babu et al. (Babu et al., 2024) developed targeted nanoparticles, administered intravenously, that demonstrated synergistic photothermal and immunotherapeutic effects against HCC, where NIR laser irradiation elevated intratumoral temperature sufficiently to induce direct tumor cell killing while simultaneously enhancing vascular permeability and triggering immunogenic cell death. The resulting adaptive immune activation produced substantial tumor regression, illustrating that the combination of thermal ablation with immune stimulation within a single nanoplatform can convert immunologically “cold” HCC tumors to “hot” phenotypes amenable to immune clearance.

### 8.4 Oral Nano-DDS as immunomodulatory adjuncts to systemic immunotherapy

The establishment of immune checkpoint inhibitor (ICI)-based regimens as standard-of-care for advanced HCC—including atezolizumab–bevacizumab and the STRIDE regimen—creates a compelling clinical rationale for oral nano-DDS that function not as standalone anticancer agents but as immunomodulatory adjuncts designed to enhance ICI response rates. This paradigm shift—from oral nano-DDS as primary cytotoxic delivery vehicles to oral nano-DDS as immune microenvironment modulators—leverages the unique strengths of oral delivery while addressing the most significant clinical limitation of current HCC immunotherapy: the approximately 70% of patients who do not achieve objective responses (Greten et al., 2023; Llovet et al., 2022).

Three mechanistically distinct immunomodulatory strategies are particularly suited to oral nano-DDS implementation.

- Gut microbiome remodeling to enhance ICI efficacy. Emerging clinical evidence has established the gut microbiome as a critical determinant of ICI response across multiple tumor types, with specific bacterial taxa (notably Akkermansia muciniphila, Faecalibacterium prausnitzii, and Bifidobacterium spp.) associated with favorable immunotherapy outcomes (Routy et al., 2018). In the specific context of HCC, a prospective study by Zheng et al. demonstrated that gut microbiome composition at baseline predicted response to anti-PD-1 immunotherapy, with responders harboring significantly higher diversity and enrichment of *Lactobacillus* and Bifidobacterium species (Zheng et al., 2019). Oral nano-DDS incorporating prebiotic polysaccharides (as discussed in Section 10.3) or engineered probiotics (Section 7.3) could serve as oral immunomodulatory adjuncts administered concurrently with systemic ICIs, with the explicit therapeutic goal of converting the gut microbiome from a non-responder to a responder phenotype. This strategy directly exploits the oral–hepatic axis: microbiome-derived metabolites, particularly SCFAs and secondary bile acids, are transported via the portal vein to the liver, where they modulate hepatic dendritic cell maturation, Kupffer cell polarization, and the balance between effector and regulatory T cells in the HCC immune microenvironment (Jia et al., 2018).
- Oral STING agonist delivery for innate immune activation: mechanistic rationale and an unmet research direction. This subsection treats oral STING-agonist nano-DDS as a forward-looking research direction rather than as an established preclinical modality. The cGAS-STING pathway is a major regulator of innate immunity in cancer and has emerged as a pivotal target for converting immunologically “cold” HCC tumours to “hot” phenotypes amenable to ICI therapy (Corrales et al., 2017). At least ten STING-pathway agents—including ADU-S100/MIW815, ulevostinag/MK-1454, E7766, BMS-986301, BI 1387446, SB11285, GSK3745417, IMSA101, SNX281 and XMT-2056—have entered early-phase oncology trials, but two practical barriers have limited their translation: cyclic-dinucleotide STING agonists are large, doubly anionic molecules with poor membrane permeability that have historically required intratumoral injection, as exemplified by the MIW815 first-in-human trial in which patients received weekly intratumoral injections of 50–6,400 μg (Meric-Bernstam et al., 2022); and systemic administration of more permeable STING agonists is associated with dose-limiting hepatic and splenic toxicity in nanoparticle formulations (Wehbe et al., 2021), an effect further exacerbated by the ubiquitous expression of STING in non-target tissues. Two parallel developments now make an oral nano-DDS approach for HCC theoretically attractive. First, the identification of MSA-2, an orally bioavailable non-nucleotide STING agonist with a tumour-microenvironment-acidity-dependent activation mechanism, demonstrates that systemic STING activation *via* the oral route is pharmacologically achievable, although that study did not include HCC models or nanoparticle formulation (Pan et al., 2020). Second, the recent demonstration by Guilbaud et al. that an oral lipid nanocapsule can durably engage the cGAS-STING axis in the gastrointestinal–portal compartment (in that case as an H-151 antagonist for inflammatory bowel disease) provides the first proof-of-feasibility for oral cGAS-STING-modulating nano-DDS (Guilbaud et al., 2025). Combined with the well-established portal-vein-mediated hepatic first-pass enrichment of orally absorbed nanoparticles, these advances suggest that an oral STING agonist nano-DDS for HCC could, in principle, concentrate the agonist in the liver before systemic distribution, achieving therapeutically relevant intrahepatic STING activation while minimising systemic inflammatory side effects. We highlight this gap as a high-priority direction for future translational research; to our knowledge, no such study has yet been published, and the present discussion should therefore be read as a mechanistic rationale rather than a summary of completed preclinical work. The sequential combination of an oral STING-agonist nano-DDS (to prime the innate immune response) followed by systemic ICI administration (to unleash the adaptive immune response) thus represents a rationally designed but as yet experimentally unrealised combination that merits dedicated investigation.
- Oral nano-DDS-mediated metabolic reprogramming of the TME. The HCC tumor microenvironment is characterized by metabolic features—including lactate accumulation, tryptophan depletion *via* IDO1/TDO2 overexpression, and adenosine enrichment—that promote immune suppression and ICI resistance (Pinato et al., 2020). Oral nano-DDS co-delivering IDO1 inhibitors (e.g., epacadostat) with conventional chemotherapeutics could achieve localized metabolic reprogramming of the hepatic TME through first-pass targeting, restoring T cell effector function and enhancing ICI sensitivity without the systemic metabolic disruption that contributed to the failure of systemic IDO1 inhibition in the ECHO-301/KEYNOTE-252 trial.

Importantly, the clinical development of oral nano-DDS immunomodulatory adjuncts could be substantially accelerated by leveraging the existing clinical trial infrastructure for ICI-based HCC regimens. Add-on study designs—where the oral nano-DDS is evaluated as an addition to the standard-of-care ICI regimen—require smaller sample sizes and shorter timelines than first-line monotherapy trials, providing a pragmatic path to clinical proof-of-concept.

### 8.5 Oral nano-DDS as maintenance therapy following locoregional interventions

Transarterial chemoembolization (TACE) remains the standard of care for intermediate-stage (BCLC-B) HCC, but post-TACE recurrence rates remain high, with 1-year recurrence exceeding 50% in many series. The post-TACE setting presents a particularly compelling clinical niche for oral nano-DDS: patients have undergone successful locoregional tumor debulking and require prolonged maintenance therapy to prevent or delay recurrence—a scenario ideally suited to the non-invasive, patient-friendly oral route. Current post-TACE adjuvant strategies, including sorafenib and lenvatinib, have shown modest benefits in randomized trials, with the TACTICS trial demonstrating that sorafenib combined with TACE improved progression-free survival compared with TACE alone (Kudo et al., 2018; Kudo et al., 2022).

Oral nano-DDS could enhance the post-TACE adjuvant approach through several mechanisms: (i) improved oral bioavailability of kinase inhibitors (sorafenib, lenvatinib) through nano-encapsulation, enabling dose reduction and consequent toxicity mitigation during prolonged maintenance therapy; (ii) co-delivery of anti-angiogenic agents with immunomodulators to address the post-TACE hypoxia-driven angiogenic rebound and immune microenvironment remodeling that drive recurrence; and (iii) gut–liver axis modulation to restore the dysbiotic microbiome that is often exacerbated by repeated TACE procedures and peri-procedural antibiotic use. The temporal window following TACE—when tumor burden is minimal and the immune microenvironment is primed by TACE-induced immunogenic cell death—represents an immunologically favorable context for oral nano-DDS-mediated immunomodulation (Llovet et al., 2021b). Clinical trial designs evaluating oral nano-DDS as post-TACE maintenance therapy would benefit from the well-defined patient population, objective radiological endpoints (mRECIST), and established comparator arms (TACE alone, TACE + sorafenib) that characterize the intermediate-stage HCC clinical trial landscape.

### 8.6 Critical assessment of combination therapeutic nano-DDS

Several cross-cutting limitations apply to the combination therapeutic strategies discussed in this section. First, the vast majority of studies were conducted via intravenous administration; the additional complexity introduced by oral delivery—including differential GI stability of co-encapsulated agents, asynchronous release kinetics, and loss of synergistic drug ratios during GI transit—has not been systematically addressed. Second, the claimed synergistic effects are frequently assessed using the Chou-Talalay combination index (CI) method *in vitro*, but these values may not translate to *in vivo* contexts where pharmacokinetic factors, tumor heterogeneity, and immune microenvironment variability profoundly influence drug interactions. Third, the increased complexity of multi-component nano-DDS platforms raises significant manufacturing and regulatory challenges: each additional therapeutic component introduces additional critical quality attributes that must be controlled, characterized, and validated under GMP conditions. Finally, a notable gap in the current literature is the absence of comparison between multi-component co-delivery systems and simple sequential administration of individual nano-formulations; such comparisons are essential to demonstrate that the added manufacturing complexity of co-delivery provides genuine therapeutic benefit over simpler dosing strategies.

## 9 Magnetic nano-formulations for HCC: toward oral theranostic applications

This section examines physically driven magnetic strategies that offer an orthogonal approach to enhance hepatic drug accumulation and enable theranostic capabilities. Their oral translation faces unique challenges—including bio-corona attenuation of magnetic responsiveness, large hydrodynamic size limiting intestinal absorption, chronic iron overload risk, and undemonstrated feasibility of external magnetic guidance through the portal venous system—which should temper translational expectations. Magnetic nanoparticles (MNPs), typically composed of superparamagnetic iron oxide (Fe_3_O_4_ or γ-Fe_2_O_3_), offer unique multifunctional capabilities for HCC therapy, including external magnetic field-guided targeting, magnetic hyperthermia, MRI-based theranostics, and magnetically triggered drug release (Laurent et al., 2011).

### 9.1 Magnetically guided hepatic targeting

External magnetic fields can guide orally administered MNP-loaded nano-formulations to concentrate at the hepatic tumor site, significantly enhancing local drug accumulation beyond that achievable by passive targeting alone. Multifunctional Fe_3_O_4_-PEI@HA nanoparticles have been developed for ferroptosis-mediated HCC treatment, combining hyaluronic acid-mediated CD44 targeting with iron oxide-induced ferroptosis through ROS modulation (Liang et al., 2023). The hyaluronic acid coating provided CD44-mediated tumor targeting, while the iron oxide core enabled both magnetic guidance and ferroptosis induction through intracellular iron release. Phosphorylated galactosylated chitosan-coated magnetic nanoparticles have demonstrated multimodal targeting capability against DEN-induced HCC, combining ASGPR-mediated uptake with magnetic guidance for enhanced therapeutic efficacy (Udupi et al., 2025).

### 9.2 Magnetic hyperthermia and MRI theranostics

MNPs subjected to alternating magnetic fields (AMFs) generate localized heat through Néel relaxation and Brownian relaxation mechanisms, achieving therapeutic hyperthermia (42 °C–46 °C) that selectively damages tumor cells while sparing surrounding normal tissue (Lee et al., 2015). Furthermore, the superparamagnetic properties of SPIONs provide excellent T_2_-weighted MRI contrast, enabling real-time monitoring of nanoparticle biodistribution and treatment response—a theranostic capability that is highly valuable for image-guided HCC therapy (Van et al., 2023).

A magnetically guided theranostic nanotubular system based on halloysite nanotubes embedded with iron–platinum nanoparticles has demonstrated dual T_2_-weighted MRI contrast and magnetic hyperthermia capability for HCC treatment (Chan et al., 2024). The system demonstrated significant tumor growth inhibition with longitudinal MRI monitoring capability. Similar MRI contrast capabilities have been explored with alternative ferrite compositions (Sobhani et al., 2023). The comprehensive landscape of superparamagnetic iron oxide nanoparticles for cancer therapy, including HCC-specific applications, has been recently reviewed by Vangijzegem et al. (Xu et al., 2022). Notably, the first clinical evidence for magnetic nanoparticle-based hyperthermia in liver cancer has recently emerged, with Kraus et al. (Kraus et al., 2025) reporting that intratumoral injection, though not *via* oral administration, of iron oxide nanoparticles followed by AMF treatment stabilized metastatic disease in stage IV primary liver cancer patients, representing an encouraging early translational step for magnetic nano-DDS in HCC, though larger controlled trials are needed to confirm therapeutic benefit. Injectable hydrogel systems incorporating magnetic nanosheets have further expanded the platform diversity for magnetic hyperthermia-based HCC treatment (Gong et al., 2024).

### 9.3 Critical assessment of magnetic nano-formulations for oral HCC delivery

Despite the theranostic appeal of magnetic nano-formulations, their translation to oral HCC delivery faces formidable challenges that warrant candid assessment. The feasibility of external magnetic field guidance through the portal venous system—a complex, low-flow vascular network embedded within the liver parenchyma—has not been demonstrated, and the physical principles governing magnetic nanoparticle navigation in portal hemodynamics differ substantially from those in arterial circulation. Bio-corona formation in the GI tract may attenuate magnetic responsiveness by increasing hydrodynamic size and reducing the effective magnetic moment per unit mass. Chronic oral exposure to iron oxide nanoparticles raises concerns about hepatic iron overload—particularly relevant in HCC patients with pre-existing cirrhosis, where hepatic iron homeostasis is already compromised. Furthermore, the only clinical evidence for magnetic nanoparticle hyperthermia in liver cancer (Kraus et al., 2025) employed intratumoral injection rather than oral delivery, and the significant technological gap between these two delivery routes should not be understated. Accordingly, magnetic oral nano-DDS for HCC remain at the earliest conceptual stage, and dedicated feasibility studies addressing GI absorption of magnetic nanoparticles, portal venous magnetic guidance, and chronic hepatic iron safety are prerequisite before further preclinical development.

## 10 Natural product-loaded oral nano-formulations and gut–liver axis modulation strategies

This section addresses two conceptually related but mechanistically distinct therapeutic strategies unified by their reliance on oral delivery: natural bioactive compound-loaded nano-DDS, which primarily target HCC cells through direct pharmacological mechanisms, and gut–liver axis modulation strategies, which target the bidirectional communication between the intestinal microbiome and the liver to reshape the tumor immune microenvironment. While these approaches are presented within a single section due to their shared exploitation of the oral delivery route, readers should recognize that they operate through fundamentally different biological mechanisms and face distinct development barriers.

The strategies described in the preceding sections have primarily focused on delivering synthetic chemotherapeutic or immunomodulatory agents to HCC tumors. However, a complementary approach leverages natural bioactive compounds with inherent hepatoprotective and anticancer properties, combined with modulation of the gut–liver axis—an anatomical connection uniquely accessible *via* oral delivery. The gut–liver axis, encompassing bidirectional communication between the intestinal microbiome and the liver through the portal venous system, bile acids, and immune mediators, plays a pivotal role in HCC pathogenesis and progression (Albillos et al., 2020). Dysbiosis-associated intestinal barrier dysfunction, endotoxemia, and chronic inflammation constitute key drivers of HCC development from cirrhotic livers. The roles of gut microbiota in HCC have been comprehensively elucidated, spanning from gut dysbiosis to the emerging concept of the intratumoral microbiota that directly influences tumor biology and treatment response (Wang Y. et al., 2025). Oral nano-formulations incorporating natural bioactive compounds and/or microbiome-modulating agents represent a holistic therapeutic strategy that simultaneously targets tumor cells and restores gut–liver axis homeostasis.

### 10.1 Curcumin-loaded oral nano-DDS

Curcumin, the principal curcuminoid of *Curcuma longa*, exhibits pleiotropic anticancer activities including suppression of NF-κB signaling, induction of apoptosis, inhibition of angiogenesis, and reversal of MDR (Gupta et al., 2013). However, its clinical utility is severely limited by poor aqueous solubility of 11 ng/mL at pH 5.0, rapid hepatic metabolism, and consequently negligible oral bioavailability (<1%) (Anand et al., 2007). Nano-encapsulation has demonstrated substantial improvement in curcumin oral bioavailability. As discussed in Section 6.1, galactosylated chitosan-coated nanoparticles have demonstrated ASGPR-mediated enhancement of curcumin delivery to HCC, significantly enhancing tumor-selective accumulation (Huang et al., 2023). In a complementary approach, smart mesoporous silica nanoparticles loaded with curcumin and coated with lactobionic acid-modified carboxymethyl chitosan have achieved pH-responsive, ASGPR-targeted delivery with effective liver cancer inhibition (Man et al., 2024). Additionally, morphologically transformable peptide nanocarriers co-loaded with doxorubicin and curcumin demonstrated inhibition of both HCC growth and metastasis through synergistic combination therapy (Liu et al., 2024). The broader therapeutic potential of curcumin and its derivatives in hepatology has been comprehensively reviewed through systematic analysis of molecular targets and therapeutic implications (Esmaeli et al., 2025). However, a critical caveat warrants acknowledgment: curcumin has been classified as both a pan-assay interference compound (PAINS) and an invalid metabolic panaceas (IMPS) compound, meaning it can generate false-positive results across multiple assay types through non-specific mechanisms including fluorescence interference, aggregation-based enzyme inhibition, membrane disruption, and chemical instability. These properties raise legitimate concerns about whether the observed anticancer activities of curcumin *in vitro* reflect genuine target-specific pharmacology or assay artifacts. While nano-encapsulation may partially mitigate some of these issues by stabilizing curcumin and improving its pharmacokinetic profile, the interpretation of preclinical efficacy data for curcumin-loaded nano-DDS should be approached with appropriate caution, and future studies should incorporate rigorous controls for PAINS-associated artifacts, including orthogonal assay validation and counter-screening against curcumin-insensitive cell lines.

### 10.2 Berberine-loaded oral nano-DDS

Berberine, an isoquinoline alkaloid derived from *Coptis chinensis*, has garnered attention for its anti-HCC activity through AMPK activation, PI3K/AKT pathway inhibition, and gut microbiota modulation (Wang et al., 2020b). Extensive research has comprehensively reviewed the potential of berberine in the treatment of HCC and other liver diseases, including NAFLD and NASH, highlighting its multifaceted hepatoprotective and anticancer mechanisms (Cervello et al., 2024). Specifically, berberine-loaded albumin nanoparticles have demonstrated the ability to reverse aflatoxin B1-induced liver hyperplasia, providing evidence for the therapeutic utility of berberine nano-formulations against hepatic pre-malignant lesions (Khedr et al., 2023). Furthermore, berberine has emerged as a prominent natural compound in nanotechnology-based cancer treatments, as nanocarrier encapsulation effectively addresses its key pharmacokinetic limitations, such as poor oral bioavailability and rapid hepatic metabolism (Sun et al., 2024). The bidirectional interplay between bile acids, gut microbiota, and HCC has provided new mechanistic insights for designing nano-formulations that simultaneously modulate hepatic bile acid signaling and restore microbial homeostasis (Song et al., 2023). A critical appraisal of the berberine nano-DDS literature reveals a recurring limitation: while multiple studies demonstrate improved oral bioavailability compared with free berberine, few have employed HCC-bearing animal models, and none have reported tumor growth inhibition data from oral berberine nano-formulations in orthotopic HCC models. The extrapolation from general hepatoprotective activity to HCC therapeutic efficacy requires dedicated validation in clinically relevant models, and the claimed gut microbiota modulation effects of berberine nano-formulations should be corroborated using shotgun metagenomics rather than 16S rRNA sequencing alone, which provides limited functional resolution.

### 10.3 Prebiotic-functionalized nano-formulations and gut–liver axis strategies

Prebiotic polysaccharides including inulin, β-glucan, and fructooligosaccharides serve dual roles as microbiome-modulating agents and colon-targeted carrier materials. Their selective fermentation by beneficial colonic bacteria produces short-chain fatty acids (SCFAs), particularly butyrate, which strengthens gut barrier function, attenuates hepatic inflammation, and independently inhibits HCC proliferation through HDAC inhibition (Donohoe et al., 2012). In a pivotal study, a Trojan-horse strategy targeting the gut–liver axis was developed that modulated the gut microbiome and reshaped the tumor microenvironment for orthotopic HCC therapy (Yao et al., 2024). Notably, the enzyme-responsive astragalus polysaccharide nanoparticles (Liu et al., 2025) further exemplify this therapeutic convergence, demonstrating that enzyme-responsive colonic delivery and gut–liver axis modulation can be synergistically integrated for NAFLD-driven hepatocarcinogenesis prevention. This oral nanoplatform based on dextran-carbenoxolone conjugate achieved significant reductions in LPS-associated pathogenic bacteria while restoring beneficial gut microbiota, resulting in attenuated hepatic inflammation and enhanced antitumor immunity in an orthotopic HCC model. The gut microbiome has been increasingly recognized as a key modulator of immunotherapy response in HCC, with gut microbiome-specific nanoparticle-based therapeutics representing an emerging strategy for enhancing immune checkpoint inhibitor efficacy through microbiome remodeling (Khurana and Hartmann, 2025; Huang et al., 2025). Enterohepatic circulation-inspired nanoplatforms that leverage the natural bile acid recycling pathway have recently been developed for less-hepatotoxic HCC therapy, further illustrating the strategic exploitation of the gut–liver axis (Liang et al., 2025).

### 10.4 Critical assessment of natural product and gut–liver axis strategies

The natural product-loaded nano-DDS and gut–liver axis modulation strategies discussed above face distinct but overlapping development hurdles. For curcumin-loaded systems, the PAINS/IMPS classification raises fundamental questions about the reliability of *in vitro* efficacy data that should temper enthusiasm for clinical translation. For berberine-loaded systems, the absence of tumor growth inhibition data from oral administration in orthotopic HCC models limits the current evidence to proof-of-concept. For gut–liver axis strategies, the most significant challenge is the profound inter-individual variability in gut microbiome composition—particularly in HCC patients with cirrhosis-associated dysbiosis and portal hypertension—which may render standardized nano-formulation approaches inadequate. Future directions should include: (i) rigorous validation of natural product nano-DDS in orthotopic HCC models on fibrotic backgrounds; (ii) characterization of gut microbiome-dependent pharmacokinetic variability using multi-omics approaches; and (iii) development of adaptive formulations whose enzyme-responsive properties can be tailored to individual patient microbiome profiles, an area where emerging computational approaches may prove valuable.

## 11 Summary and comparative analysis of Nano-DDS platforms for HCC therapy

As summarized in Tables 3, 4 only a minority of representative nano-formulations have been validated via oral delivery, with gut–liver axis modulation and natural product-loaded formulations representing the most mature oral candidates. Systematic oral validation using clinically relevant orthotopic models and standardized pharmacokinetic benchmarking remains the most pressing research priority.

**TABLE 3:** Representative nano-formulations for HCC therapy discussed in this review.

Category	Nano-formulation	Therapeutic agent	Targeting strategy	Animal model	Route	Key outcome	Limitations	Ref.
pH-responsive	Tim-3 siRNA/Sor CaCO_3_ NPs	Sorafenib + Tim-3 siRNA	pH-triggered co-release	HCC mice	IV	Enhanced antitumor efficacy vs. single agents	*In vivo* immune monitoring limited	Song et al. (2022)
pH-responsive	ZIF-8@Alginate microparticles	Sorafenib	Microfluidic oral ZIF-8	HCC xenograft	**Oral**	Improved reproducibility; pH-responsive release	Subcutaneous model only	Mirshafiei et al. (2026)
pH/GSH dual	Gal-Core–Shell NPs	Anticancer drug	ASGPR + GSH-triggered	*In vitro* HCC	*In vitro*	Selective HCC cytotoxicity; dual responsiveness	No *in vivo* validation	Qu et al. (2023)
ROS/pH cascade	GA-thioketal NPs	Chemotherapeutic	Glycyrrhetinic acid + ROS	HCC mice	IV	ROS amplification + tumor growth inhibition	ROS paradox unexplored	Shi et al. (2024)
ASGPR-targeted	Gal-CS NPs	Curcumin	Galactose-ASGPR	*In vitro* HCC	*In vitro*	Enhanced hepatocyte uptake	*In vivo* data limited	Huang et al. (2023)
GPC3-targeted	GPC3-Fe_3_O_4_-GOD NPs	Catalytic therapy	GPC3 peptide	HCC mice	IV	Dual imaging + catalytic therapy	Complex preparation	Deng et al. (2022)
Biomimetic	Homologous CM NPs	Lenvatinib	Cancer cell membrane	HCC mice	IV	Enhanced chemotherapy efficacy	Membrane batch variability	Wu et al. (2023)
Exosome-based	Milk exosomes	Various drugs	Exosome transcytosis	*In vitro*/vivo	**Oral**	GI stability; efficient absorption	Scalability challenges	Li et al. (2023)
Probiotic	Engineered EcN	Neoantigens	Bacterial colonization	Multiple models	**Oral**	Potent antitumor immunity	GMO regulatory complexity	Redenti et al. (2024)
Photothermal-immuno	Targeted NPs + NIR	PTT agent + immunomodulator	Ligand-targeted + NIR	HCC mice	IV	Synergistic PTT + immune activation	Limited NIR tissue penetration	Babu et al. (2024)
Magnetic theranostic	FePt@HNT NPs	Hyperthermia	Magnetic + MRI	HCC cells	*In* *vitro*	T_2_ MRI contrast + magnetic hyperthermia	Chronic IONP accumulation risk	Chan et al. (2024)
Curcumin NPs	GC-MSN-Cur	Curcumin	LA-ASGPR + pH	HCC mice	**Oral**	pH-responsive liver-targeted delivery	Curcumin monotherapy limited	Man et al. (2024)
Gut–liver axis	Dextran-CBX NPs	Carbenoxolone	Gut microbiota modulation	Orthotopic HCC	**Oral**	Microbiome remodeling + antitumor immunity	SCFA contribution not deconvoluted	Yao et al. (2024)

Route column indicates the actual route of administration employed in the cited study. Bold entries highlight the administration route: Oral = validated via oral delivery; IV, intravenous; *In vitro* = cell-based evaluation only. Studies administered via non-oral routes are included for their mechanistic insights relevant to oral nano-DDS, design.

Abbreviations: ASGPR, asialoglycoprotein receptor; CBX, carbenoxolone; CM, cell membrane; GA, glycyrrhetinic acid; GC, galactosylated chitosan; HNT, halloysite nanotube; LA, lactobionic acid; MRI, magnetic resonance imaging; MSN, mesoporous silica nanoparticle; NPs, nanoparticles; PTT, photothermal therapy; SCFA, short-chain fatty acid.

**TABLE 4:** Semi-quantitative comparison of oral nano-DDS platform strategies for HCC therapy.

Platform strategy	Particle size range (nm)	Typical drug loading (% w/w)	Reported oral bioavailability enhancement (vs. Free drug)	GI stability evidence	Oral route validation status	Key advantage	Primary development barrier
Polymeric NPs (PLGA, chitosan)	80–250	5–15	2–8-fold	Moderate (pH-dependent)	Multiple oral studies	Regulatory precedent (FDA-approved polymers)	Limited drug loading; burst release
Lipid-based NPs (SLN, NLC, SNEDDS)	50–300	10–50	3–15-fold	Variable (lipase-sensitive)	Several oral studies	High loading; lymphatic transport	Food effects; lipase degradation
Inorganic NPs (MSN, ZIF-8)	30–200	15–40	2–10-fold	Good (pH-dependent dissolution)	Limited oral studies	High surface area; stimulus-responsive pores	Chronic toxicity; tissue accumulation
Cell membrane-coated NPs	100–250	5–15 (core-dependent)	Not reported (oral)	Unknown (GI conditions)	No oral studies	Immune evasion; homotypic targeting	Membrane GI stability; scalability
Exosome-based systems	30–150	1–10	3–5-fold (estimated)	Moderate (milk-derived)	Early oral studies	Biocompatibility; barrier crossing	Purification scalability; batch variability
Probiotic-based systems	N/A (bacterial)	N/A (biosynthesized)	N/A (*in situ* production)	Inherent (GI colonization)	Oral by design	GI colonization; *in situ* production	GMO regulation; biosafety; immune risk
Magnetic NPs	10–100 (core)/100–300 (coated)	5–20	Not reported (oral)	Unknown	No oral studies	Theranostic capability; magnetic guidance	Portal magnetic guidance feasibility; iron overload
Gut–liver axis modulators	100–300	Variable	N/A (microbiome modulation)	Good (designed for GI)	Oral by design	Addresses HCC etiology; immune remodeling	Microbiome inter-individual variability

Bioavailability enhancement values are approximate ranges compiled from representative studies discussed in this review and should be interpreted as indicative rather than definitive, given the heterogeneity of experimental conditions, animal models, drug payloads, and measurement methodologies across studies. “Oral Route Validation Status” reflects the current preclinical evidence base as assessed through the route-of-administration audit described above.

## 12 Computational approaches and artificial intelligence: emerging opportunities for oral Nano-DDS design

The design and optimization of oral nano-DDS for HCC therapy have traditionally relied on empirical formulation screening. Computational modeling and artificial intelligence (AI) may accelerate candidate selection, but this section should be read as an opportunity map rather than evidence of established oral HCC nano-DDS performance (Chou et al., 2025; Agrahari et al., 2024). No AI-optimized oral nano-DDS for HCC has yet been clinically validated, and most cited computational work addresses parenteral nanomedicine, lipid nanoparticles, or general formulation development. For oral HCC applications, the most immediate uses are practical and testable: predicting nanoparticle stability across the GI pH gradient, modeling mucus and bile-salt interactions, estimating portal *versus* lymphatic transport, supporting PBPK-based dose projection, and prioritizing formulations for orthotopic/fibrotic model testing.

### 12.1 Machine learning-driven formulation optimization

Machine learning (ML) algorithms—including random forests, gradient boosting, support vector machines, and neural networks—have demonstrated notable success in predicting nanoparticle physicochemical properties, including size, polydispersity, zeta potential, and encapsulation efficiency, from formulation variables, including polymer composition, drug-to-excipient ratio, and preparation method parameters (Bannigan et al., 2021). For oral nano-DDS specifically, ML models can predict oral bioavailability enhancement by integrating molecular descriptors of the drug, nanocarrier composition, and GI physiological parameters, potentially enabling *in silico* screening of formulation candidates before costly *in vivo* evaluation. Recent advances have demonstrated that ML-driven approaches can dramatically accelerate lipid nanoparticle optimization. Combinatorial chemistry coupled with ML achieved screening speeds orders of magnitude faster than traditional approaches (Li B. et al., 2024), while deep learning platforms such as AGILE showed remarkable predictive accuracy for mRNA delivery (Xu et al., 2024). Generative models have enabled screening of approximately 20 million ionizable lipids, with all six second-iteration candidates matching or outperforming benchmark lipids MC3 and SM-102 (Wang W. et al., 2024). Industry perspectives have further endorsed these ML-driven approaches for formulation and process development (Dorsey et al., 2024). The development of curated, publicly available datasets of oral nanoparticle pharmacokinetic outcomes would substantially accelerate progress in this area.

### 12.2 Molecular dynamics simulation for mucus–nanoparticle interactions

Coarse-grained and all-atom molecular dynamics (MD) simulations provide atomic-level insight into nanoparticle–mucin interactions, lipid bilayer permeation, and receptor–ligand binding thermodynamics that are difficult to characterize experimentally (Durrant and McCammon, 2011). Recent work integrating ML with MD simulations has demonstrated the potential for computationally guided design of nanoparticle surface chemistries optimized for both mucus penetration and cellular uptake (Jahandoost et al., 2024). These computational approaches can guide the rational selection of surface coatings, ligand densities, and particle geometries that optimize mucus penetration and epithelial transcytosis. Integration of MD simulation with experimental validation in a closed-loop design framework represents a promising paradigm for accelerating oral nano-DDS development.

### 12.3 Digital twins and physiologically based pharmacokinetic modeling

Physiologically based pharmacokinetic (PBPK) modeling of oral nanoparticle absorption, distribution, metabolism, and excretion enables *in silico* prediction of clinical pharmacokinetics from preclinical data, facilitating first-in-human dose selection and reducing clinical attrition rates (Dogr et al., 2019; Ozbek et al., 2024). AI-assisted PBPK and deep neural network models have demonstrated high predictive accuracy for nanoparticle tumor delivery in mice, with reported R^2^ values of 0.83 and 0.92 respectively (Chou et al., 2023; Lin et al., 2022), establishing a computational framework for translational dose optimization. The construction of “digital twin” models that integrate individual patient parameters, including hepatic function, portal hemodynamics, tumor burden, and gut microbiome composition, with nano-DDS pharmacokinetic models holds promise for personalized dose optimization in HCC patients with heterogeneous disease presentations (Bordukova et al., 2024). The convergence of AI-driven drug design with nanotechnology and liver cancer biology represents a rapidly maturing field, with comprehensive frameworks for rational nanomedicine design in HCC increasingly within reach (Bhange and Telange, 2025).

## 13 Bottlenecks and future directions

Despite the considerable progress summarized in this review, the clinical translation of oral nano-DDS for HCC therapy remains in its nascent stages, facing several formidable challenges that must be systematically addressed.

### 13.1 Scalability and manufacturing reproducibility

The multi-step synthesis, functionalization, and quality control of complex oral nano-formulations present significant manufacturing challenges. Batch-to-batch variability in particle size distribution, surface ligand density, drug encapsulation efficiency, and release kinetics must be minimized to meet regulatory standards. Microfluidic continuous-flow synthesis, which offers superior reproducibility and scalability compared with conventional batch methods, represents a promising manufacturing solution (Valencia et al., 2012). The development of quality-by-design (QbD) frameworks incorporating process analytical technology (PAT) and real-time release testing (RTRT) will be essential for robust large-scale production (Birla et al., 2025; Buya et al., 2024). Encouragingly, recent advances in impingement jet and flash nanoprecipitation technologies have demonstrated the capacity to produce polymeric nanoparticles at kilogram-per-hour scales with coefficient of variation in particle size below 5%, suggesting that manufacturing scalability need not be an insurmountable barrier for relatively simple nano-formulations. The establishment of ICH-compliant specifications for critical quality attributes (CQAs) of oral nano-formulations—including particle size, polydispersity, surface charge, drug loading, enteric coating integrity, and dissolution under biorelevant conditions—will be essential for standardizing manufacturing processes across the field.

### 13.2 Safety and chronic toxicity considerations

While most nanomaterials employed in oral HCC DDS demonstrate acceptable acute biocompatibility in preclinical models, the long-term toxicological consequences of chronic oral nanoparticle exposure remain insufficiently characterized (Havelikar et al., 2024; Bondarenko et al., 2021). Specific concerns include the accumulation of non-biodegradable inorganic nanomaterials, including silica and metal oxides, in hepatic and splenic tissues, the immunological consequences of chronic nanoparticle–gut microbiome interactions, and the potential genotoxicity of nanomaterials with high surface reactivity (Domingues et al., 2022). Comprehensive chronic toxicity studies, including 6–12-month repeated-dose oral toxicity, reproductive toxicity, and carcinogenicity assessments, will be prerequisite for regulatory approval. Furthermore, the potential for nanoparticles to exacerbate pre-existing liver disease, including cirrhosis and portal hypertension, in the HCC patient population—which overwhelmingly presents with compromised hepatic function—has been almost entirely neglected in current preclinical safety assessments and represents a critical knowledge gap.

A systematic framework for evaluating the safety of oral nano-DDS in the context of HCC therapy should encompass four interconnected domains, each requiring methodology tailored to the unique pathophysiology of the target patient population.

- Hepatotoxicity assessment in pre-damaged liver models. The overwhelming majority of HCC patients present with underlying chronic liver disease (cirrhosis, fibrosis, or steatohepatitis), yet virtually all preclinical nanotoxicology studies evaluate nanoparticle safety in animals with healthy livers. This represents a critical disconnect: the compromised regenerative capacity, altered sinusoidal architecture, and dysregulated immune homeostasis of the cirrhotic liver may substantially amplify nanoparticle-induced hepatotoxicity. Studies employing conventional hepatotoxicity endpoints (ALT, AST, bilirubin) in healthy animals may therefore significantly underestimate the hepatotoxic potential of oral nano-DDS in the target patient population. We recommend that chronic toxicity studies of oral nano-DDS for HCC routinely include a fibrotic liver cohort (e.g., CCl4-or thioacetamide-induced fibrosis models) alongside healthy controls, with expanded endpoints including hepatic stellate cell activation markers (α-SMA, collagen I/III deposition), sinusoidal endothelial cell function (hyaluronic acid clearance capacity), and quantitative assessment of fibrosis progression using second harmonic generation microscopy or collagen proportionate area measurement (Ramachandran et al., 2019).
- Immunotoxicity with focus on hepatic immune tolerance. The liver maintains a unique state of immune tolerance mediated by Kupffer cells, liver sinusoidal endothelial cells, and hepatic stellate cells. Chronic nanoparticle exposure may disrupt this tolerogenic milieu, potentially triggering autoimmune hepatitis-like reactions or, conversely, further deepening the immunosuppressive environment that facilitates HCC progression. Specific immunotoxicity endpoints should include: Kupffer cell phenotyping (M1/M2 polarization markers), assessment of nanoparticle-induced trained immunity or immune tolerance in hepatic macrophages, quantification of liver-resident NK cell and NKT cell populations, and evaluation of hepatic T cell exhaustion markers (PD-1, TIM-3, LAG-3 expression on intrahepatic lymphocytes) (Robinson et al., 2016). These assessments are particularly critical for inorganic nanoplatforms, where chronic tissue accumulation creates sustained immune stimulation.
- Gut microbiome perturbation assessment. Chronic oral nanoparticle administration inevitably exposes the intestinal microbiome to sustained nanoparticle contact. Emerging evidence from the food nanotechnology field indicates that oral exposure to TiO2 nanoparticles, SiO2 nanoparticles, and ZnO nanoparticles can induce significant alterations in gut microbiome composition, intestinal barrier function, and mucosal immune homeostasis (Lamas et al., 2020). For HCC patients, in whom gut dysbiosis is already a pathogenic driver, nanoparticle-induced microbiome perturbation could exacerbate the pro-tumorigenic gut–liver axis signaling. Safety evaluation protocols for oral nano-DDS should therefore incorporate longitudinal microbiome profiling (ideally using shotgun metagenomics rather than 16S rRNA amplicon sequencing, to capture functional changes), intestinal permeability assessment (serum LPS, intestinal fatty acid-binding protein), and fecal SCFA quantification as standard endpoints.
- Hierarchical in vitro-to-in vivo safety assessment strategy. Given the resource-intensive nature of comprehensive *in vivo* toxicity studies, we propose a tiered screening approach. The first tier employs high-throughput *in vitro* screening using human-relevant models: primary human hepatocyte spheroids or HepaRG cells for hepatotoxicity, human intestinal organoids for GI toxicity, and THP-1 macrophage-derived Kupffer cell models for immunotoxicity. The second tier employs organ-on-chip platforms—specifically, gut–liver-on-chip systems that recapitulate the oral-to-hepatic delivery cascade—for integrated toxicokinetic assessment under physiologically relevant flow conditions (Herland et al., 2020). Only candidates passing both *in vitro* tiers would proceed to the third tier of conventional *in vivo* toxicity studies, thereby reducing animal usage while improving the human relevance of safety assessments. The recent development of multi-organ-on-chip platforms incorporating gut, liver, and immune compartments provides a particularly promising tool for evaluating the systemic toxicological consequences of chronic oral nanoparticle exposure in a human-relevant *in vitro* system.

### 13.3 Preclinical model limitations

The majority of studies reviewed herein employed subcutaneous xenograft or chemically induced HCC models in rodents, which inadequately recapitulate the heterogeneity, fibrotic microenvironment, and immune landscape of human HCC arising on cirrhotic liver backgrounds (Brown et al., 2018). Orthotopic patient-derived xenograft (PDX) models, genetically engineered mouse models (GEMMs) of HCC, such as the Mst1/Mst2 double knockout and albumin-Cre driven oncogene models, and humanized liver mouse models should be prioritized in future preclinical evaluations to better predict clinical efficacy (Connor et al., 2018). Additionally, the emerging field of liver-on-a-chip and gut–liver-on-a-chip microfluidic platforms offers an opportunity to evaluate oral nano-DDS performance across the full GI-to-liver delivery cascade in a human-relevant *in vitro* system, potentially reducing reliance on animal models while providing mechanistic insights into species-specific differences in nanoparticle absorption and hepatic disposition (Table 5).

**TABLE 5:** Recommended model-validation ladder for oral HCC nano-DDS, emphasizing what each experimental tier can and cannot justify.

Validation tier	Minimum model or assay	What it can support	What it cannot support alone
Tier 1: *in vitro* screening	HCC cells plus normal hepatocytes; intestinal epithelial models; mucus interaction assays	Initial cytotoxicity, uptake, GI-barrier compatibility, and ligand-binding feasibility	*In vivo* oral absorption, hepatic exposure, tumor targeting, or safety
Tier 2: oral PK platform testing	Healthy rodents with oral dosing; portal and systemic sampling; liver/spleen biodistribution	Oral absorption, first-pass exposure, tissue distribution, and formulation stability	Therapeutic efficacy in clinically relevant HCC
Tier 3: preliminary efficacy	Subcutaneous HCC xenograft with oral dosing	Initial antitumor activity and dose-ranging feasibility	Orthotopic tumor targeting, cirrhosis effects, immune microenvironment relevance
Tier 4: disease-relevant efficacy	Orthotopic HCC models, preferably with fibrotic or cirrhotic liver background	Clinically relevant tumor accumulation, efficacy, portal-flow constraints, and liver toxicity	Full human predictiveness or long-term safety
Tier 5: translational bridge	Humanized liver models, patient-derived organoids/PDX, gut–liver-on-chip systems	Patient heterogeneity, species differences, and individualized response prediction	Regulatory safety package without confirmatory *in vivo* toxicology

### 13.4 Personalized medicine and companion diagnostics

The substantial inter-patient heterogeneity in HCC molecular subtype, hepatic function according to the Child–Pugh classification, portal hypertension severity, and gut microbiome composition necessitates a personalized approach to oral nano-DDS therapy. Integration of liquid biopsy-based companion diagnostics, such as circulating tumor DNA and exosomal biomarkers, with adaptive nano-formulation design—where targeting ligands and therapeutic payloads are selected based on individual tumor molecular profiling—represents the future paradigm of precision oral nanomedicine for HCC (Ng et al., 2018). The convergence of AI-driven drug design, patient-derived organoid drug sensitivity testing, and modular nanocarrier platforms capable of rapid customization could enable a truly personalized oral nano-DDS pipeline within the coming decade. Shan et al. (Shan et al., 2024) have outlined rational strategies for improving the efficiency of nanomedicine design and discovery, emphasizing the importance of systematic parameter optimization guided by computational models rather than empirical experimentation.

### 13.5 Quantitative pharmacokinetic benchmarking

A pervasive limitation across the oral nano-DDS literature for HCC is the paucity of standardized, quantitative pharmacokinetic data that would enable meaningful cross-study comparisons. Specifically, the following critical metrics are rarely reported in a standardized manner: (i) absolute oral bioavailability (F%) determined by comparison of oral and intravenous AUC values for the same formulation; (ii) hepatic first-pass extraction ratios and portal-to-systemic drug concentration gradients; (iii) tumor-to-liver and tumor-to-plasma drug concentration ratios at pharmacologically relevant time points; and (iv) dose-normalized tumor growth inhibition rates that enable comparison across studies employing different drug doses and dosing frequencies. The absence of these standardized metrics makes it exceedingly difficult to rank-order different nano-DDS strategies by actual oral delivery efficiency or to identify the design parameters most critical for clinical performance. We recommend that future preclinical studies of oral nano-DDS for HCC routinely report: oral bioavailability (F%), AUC_0-24_ with dose normalization, hepatic drug accumulation relative to other organs, and tumor growth inhibition normalized to administered dose. The adoption of such reporting standards would substantially accelerate the identification of lead candidates for clinical translation and enable the development of predictive structure–activity relationships for oral nano-DDS design.

To illustrate this limitation without implying a formal meta-analysis, the oral studies identified in this review were evaluated as an evidence audit rather than as a pooled quantitative dataset. Reporting was highly heterogeneous: some studies provided absolute oral bioavailability, others reported only relative bioavailability against a crude suspension, and many did not distinguish intact nanoparticle-associated drug from prematurely released drug. Therefore, reported fold-enhancement values should be interpreted as formulation-specific screening signals rather than as directly comparable estimates of clinical advantage.

The most clinically informative metrics for HCC targeting—portal-to-systemic exposure gradients, tumor-to-normal-liver AUC ratios, Kupffer-cell sequestration, and comparison with marketed oral formulations—remain inconsistently reported. This gap is particularly important because an increase in total liver concentration does not necessarily indicate tumor-selective delivery; it may instead reflect uptake by normal hepatocytes, sinusoidal endothelium, or hepatic macrophages. Future studies should therefore report compartment-resolved exposure data whenever feasible.

These observations highlight the need for standardized pharmacokinetic study designs. We specifically recommend the adoption of the following minimal reporting standards for future oral nano-DDS studies: (i) absolute oral bioavailability (F%) determined using IV administration of the same formulation or a validated IV reference; (ii) portal vein drug concentration measurement at designated time points (at minimum, Tmax and 2× Tmax) to quantify first-pass hepatic exposure; (iii) liver-to-plasma and tumor-to-liver AUC ratios as standardized metrics for hepatic targeting efficiency; (iv) comparison with the marketed oral formulation (where available), not merely with free drug suspension; and (v) assessment under both fasted and fed conditions to characterize food effect magnitude. The adoption of these standards, modeled on established bioanalytical method validation guidelines (ICH M10), would enable the first meaningful cross-platform comparisons and accelerate identification of lead candidates.

### 13.6 Practical characterization package, regulatory pathways, and clinical translation

The regulatory discussion should be translated into an operational development package. For oral HCC nanomedicines, a minimum preclinical dossier should include: (i) physicochemical characterization of particle size distribution, polydispersity index, morphology, zeta potential, drug loading, encapsulation efficiency, ligand density, and batch reproducibility; (ii) release and stability testing in simulated gastric fluid, simulated intestinal fluid, FaSSIF, and FeSSIF under both fasted and fed conditions; (iii) assessment of mucus penetration, digestive enzyme exposure, bile-salt interactions, and GI bio-corona formation; (iv) oral pharmacokinetics including absolute bioavailability, portal-to-systemic concentration gradients, liver-to-plasma AUC, tumor-to-normal-liver AUC, and intact nanoparticle versus released drug quantification; (v) repeat-dose toxicology with hepatotoxicity endpoints in healthy and fibrotic/cirrhotic liver models; (vi) immunogenicity, complement activation, gut microbiome perturbation, and liver/spleen accumulation assessment; and (vii) benchmarking against clinically used oral formulations when available. The seven domains, with their purpose and the specific endpoints expected for each, are summarized in Table 6. This practical checklist responds directly to the central development gap: without standardized characterization and clinically meaningful comparators, formulation-level improvements cannot be reliably distinguished from true therapeutic advantages.

**TABLE 6:** Minimum characterization and translational evidence package recommended for oral HCC nano-DDS before IND-enabling development.

Domain	Minimum recommended data package	Purpose
Physicochemical quality attributes	Particle size distribution, PDI, morphology, zeta potential, drug loading, encapsulation efficiency, residual solvents, ligand density, batch-to-batch variability	Defines critical quality attributes and manufacturing reproducibility
Biorelevant GI performance	Release and stability in SGF/SIF plus FaSSIF/FeSSIF, mucus interaction, digestive enzyme/bile-salt stability, bio-corona characterization, fed/fasted dissolution	Tests whether the formulation can survive oral transit under realistic conditions
Pharmacokinetics and biodistribution	Absolute oral bioavailability, portal-to-systemic concentration ratio, liver-to-plasma AUC, tumor-to-normal-liver AUC, intact nanoparticle versus released drug quantification	Separates true hepatic/tumor targeting from general exposure enhancement
Safety and repeat-dose toxicology	28-day and longer repeat-dose toxicity, hepatotoxicity in fibrotic/cirrhotic liver models, immunogenicity, gut microbiome perturbation, liver/spleen accumulation, genotoxicity where relevant	Addresses chronic oral exposure and compromised hepatic reserve in HCC patients
Clinical benchmarking	Comparison with free drug, matched non-responsive nanocarrier, marketed oral formulation where available, and clinically relevant dose schedules	Prevents overstatement of benefit from weak comparators
Model relevance	Orthotopic HCC, fibrotic/cirrhotic background, immune-competent or humanized models, and post-TACE or ICI-combination settings where appropriate	Improves translation beyond subcutaneous xenograft efficacy

Clinically, no oral nano-formulation specifically designed for HCC therapy has yet entered a mature clinical development pathway based on the trial-registry evidence summarized in this review. However, adjacent precedents are informative. Oral lipid or nanocrystal-based products in other indications demonstrate that oral nanomedicine can be commercialized when the formulation provides reproducible exposure and manufacturable quality attributes. Oncology-relevant examples such as oral paclitaxel nano-formulations and oral taxane strategies combined with efflux modulation provide useful regulatory and trial-design templates, but they do not remove the need for HCC-specific validation in patients with impaired hepatic reserve, portal hypertension, and heterogeneous tumor biology.

### 13.7 Clinical translation roadmap: a platform-stratified perspective

To move beyond general calls for “more translational research,” we propose a platform-stratified clinical translation roadmap that categorizes oral nano-DDS strategies by their current technology readiness level (TRL) and identifies the specific milestone studies required to advance each platform toward investigational new drug (IND)-enabling status.

Tier 1 — Near-term candidates (TRL 4–5; IND-enabling studies feasible within 3–5 years). Polymeric nanoparticles (PLGA, chitosan) and lipid-based nanocarriers (SLN, NLC, SNEDDS) have the clearest near-term path because they benefit from established excipient familiarity, scalable manufacturing options, and oral-formulation precedents such as nanocrystal or lipid-based products. For these platforms, the critical path to clinical translation requires: (a) GMP-compatible manufacturing with demonstrated batch-to-batch consistency; (b) 28-day and longer repeated-dose oral toxicity studies in two species with comprehensive hepatotoxicity endpoints, including fibrotic or cirrhotic liver models; (c) comparative oral pharmacokinetic studies benchmarked against free drug and marketed oral formulations where available; (d) fed/fasted PK and food-effect assessment; and (e) efficacy demonstration in at least one orthotopic HCC model on a fibrotic background.

Importantly, instructive precedent exists from adjacent therapeutic areas. The oral paclitaxel nano-formulation (Liporaxel®/DHP107), an oral lipid nanoparticle formulation of paclitaxel, received regulatory approval in South Korea for gastric cancer in 2016 and has undergone Phase III evaluation, demonstrating that oral nano-DDS for oncology applications can navigate the regulatory pathway (Kang et al., 2018). Similarly, oral docetaxel co-administered with the P-gp inhibitor ritonavir (ModraDoc006/r) has completed Phase II trials in multiple solid tumors, providing a regulatory roadmap for oral chemotherapeutic nano-formulations that incorporate efflux pump inhibition strategies. These precedents offer directly relevant regulatory and clinical design templates for oral nano-DDS in HCC, yet neither has been discussed in the context of liver cancer-targeted oral nanomedicine.

Tier 2 — Medium-term candidates (TRL 2–3; requiring 5–8 years of dedicated development). Inorganic nanoplatforms (MSN, ZIF-8), stimulus-responsive multi-component systems, and exosome-based platforms require additional fundamental characterization before IND-enabling studies become appropriate. Priority milestones include: (a) comprehensive chronic oral toxicity evaluation (6–12 months) with specific attention to hepatic and splenic tissue accumulation kinetics; (b) development of scalable purification protocols (for exosomes) or continuous-flow synthesis methods (for inorganic platforms) capable of meeting GMP requirements; (c) systematic characterization of bio-corona formation and its impact on targeting ligand functionality under biorelevant GI conditions; and (d) establishment of validated bioanalytical methods for quantifying intact nanoparticle versus released drug concentrations in portal and systemic circulation.

Tier 3 — Long-term exploratory candidates (TRL 1–2; requiring >8 years). Cell membrane-coated nanoparticles, probiotic-based delivery systems, and magnetic oral theranostic platforms remain at the proof-of-concept stage for oral HCC delivery and face fundamental scientific questions that must be resolved before preclinical development can meaningfully proceed. For cell membrane-coated systems, demonstrating membrane coating integrity after GI transit is prerequisite. For probiotic systems, establishing predictable pharmacokinetic behavior that meets the dose–exposure relationships required for anticancer therapy represents a fundamental challenge distinct from their established role in microbiome modulation. For magnetic systems, demonstrating the physical feasibility of portal venous magnetic guidance is an essential first step.

This tiered framework is intended to guide resource allocation and collaborative prioritization across the field, moving the discourse from “what is possible in principle” to “what is achievable within defined timelines with identified milestone experiments.”

To make the roadmap operational, candidate formulations should be advanced only when they meet predefined go/no-go criteria rather than broad qualitative claims of improved delivery. The most important decision points are summarized below.

Together, Tables 6-8 convert the review from a descriptive catalogue of nano-DDS concepts into a route-specific development framework. This structure clarifies which claims are currently supported by direct oral evidence, which remain mechanistic extrapolations from non-oral studies, and which experiments are required before oral HCC nano-DDS can be considered clinically ready.

**TABLE 7:** Clinically meaningful comparator framework for interpreting oral nano-DDS performance claims.

Comparator	Appropriate use	Interpretive limitation
Free drug suspension	Early formulation screening when no marketed formulation exists	May exaggerate fold-improvement because poor dissolution of the suspension is an artificially weak benchmark
Solubilized drug solution	Estimating absorption ceiling and separating dissolution effects from permeability effects	May not represent clinically feasible dosing conditions
Marketed oral product (e.g., approved tablet/capsule when available)	Most clinically meaningful benchmark for oral bioavailability, safety, and dose reduction claims	Requires matched dose, fed/fasted state, and validated bioanalytical comparison
Non-targeted version of the same nanocarrier	Testing whether the targeting ligand adds value	Does not test whether the platform beats current clinical formulation
Non-responsive version of the same nanocarrier	Testing whether pH/ROS/GSH/enzyme/thermal responsiveness adds value	Requires matched size, loading, release kinetics, and surface chemistry to avoid confounding

**TABLE 8:** Practical go/no-go criteria for advancing oral HCC nano-DDS candidates toward IND-enabling development.

Decision domain	Advance criterion	Stop or redesign signal
Oral delivery performance	Demonstrated stability in SGF/SIF and FaSSIF/FeSSIF, acceptable fed/fasted variability, and measurable systemic or portal exposure	Rapid GI degradation, high food-effect variability, or no exposure advantage over relevant comparator
Clinical comparator value	Non-inferior or superior exposure compared with marketed oral formulation, or clear dose/toxicity reduction rationale	Improvement only versus crude suspension without advantage over clinical formulation
Hepatic/tumor targeting	Liver-to-plasma and tumor-to-normal-liver AUC ratios reported with intact nanoparticle/released-drug distinction	Only total liver accumulation reported, or uptake dominated by Kupffer-cell sequestration
Model relevance	Efficacy confirmed in oral dosing studies using orthotopic and preferably fibrotic/cirrhotic HCC models	Efficacy supported only by subcutaneous xenografts or *in vitro* assays
Safety readiness	Repeat-dose oral toxicity, hepatotoxicity in compromised liver models, immunogenicity, complement activation, microbiome perturbation, and liver/spleen accumulation assessed	Acute toxicity only, no chronic exposure assessment, or evidence of fibrosis/microbiome worsening
Manufacturing feasibility	Scalable process with reproducible CQAs: size, PDI, zeta potential, loading, release, ligand density, sterility/bioburden, and stability	Complex batch process with unstable CQAs or poorly characterized biomimetic components

### 13.8 Limitations of the present review

Although the revised manuscript uses a structured search strategy, route-of-administration audit, and evidence-level framework, it should not be interpreted as a full systematic review or quantitative meta-analysis. The heterogeneity of nanocarrier composition, payload dose, animal model, comparator formulation, sampling time, and pharmacokinetic endpoint precludes reliable pooled effect estimates. In addition, some original reports did not fully specify route details, comparator composition, intact-particle quantification, or tumor-versus-normal-liver biodistribution methods; in such cases, this review applies conservative interpretation. These limitations justify the emphasis on evidence mapping, route-specific wording, clinically meaningful comparators, and go/no-go development criteria rather than simple ranking of platforms by reported efficacy.

## 14 Conclusions and outlook

Oral nanomaterial-based drug delivery systems remain a promising but incompletely validated strategy for HCC therapy. Their main appeal lies in patient-friendly administration, potential protection of unstable or poorly soluble agents during GI transit, possible enrichment of hepatic exposure through portal absorption, and opportunities for combination therapy or gut–liver-axis modulation. However, the present evidence does not yet justify broad claims of reliable tumor-selective oral delivery in human HCC. The field remains dominated by parenteral mechanistic studies, *in vitro* proof-of-concept systems, and subcutaneous xenograft models, whereas direct oral validation in orthotopic, fibrotic, cirrhotic, and immune-relevant HCC settings remains limited.

A balanced interpretation therefore requires several cautions: (i) portal venous exposure is a potential advantage, not a guarantee of HCC-selective targeting; (ii) stimulus-responsive release depends on heterogeneous and patient-specific tumor microenvironmental triggers; (iii) ligand-targeted systems may fail if receptor expression is lost, masked by bio-corona formation, or redirected to normal liver or macrophage compartments; (iv) bioavailability gains relative to crude free-drug suspension may not exceed the performance of marketed oral formulations; and (v) chronic safety, immunogenicity, gut microbiome effects, and liver/spleen accumulation remain insufficiently characterized for long-term oral administration.

Future progress should prioritize a smaller number of clinically realistic experiments rather than repeated proof-of-concept demonstrations. Key priorities include standardized oral PK reporting, fed/fasted biorelevant dissolution testing, marketed-formulation comparators, matched non-responsive nanocarrier controls, portal-vein and tumor-to-normal-liver exposure measurements, orthotopic/fibrotic HCC models, repeat-dose toxicology in compromised liver settings, and clear evidence-level labeling of oral versus non-oral studies. These steps would allow the field to move from attractive design concepts toward evidence-based selection of candidates suitable for IND-enabling development.

In summary, oral nano-DDS for HCC should be viewed as an emerging translational opportunity rather than a mature therapeutic class. Its clinical value will depend on whether future studies can demonstrate reproducible oral absorption, clinically meaningful hepatic or tumor exposure beyond existing oral formulations, acceptable long-term safety in diseased liver, and efficacy in models that recapitulate the anatomical and immunological complexity of human HCC.
